# Mass spectrometric profiling of microbial polysaccharides using laser desorption/ionization – time-of-flight (LDI-TOF) and liquid chromatography-mass spectrometry (LC-MS): a novel method for structural fingerprinting and derivatization

**DOI:** 10.3389/fcimb.2025.1658802

**Published:** 2025-10-03

**Authors:** Lucia Dadovska, Veronika Paskova, Petr Novak, Jaroslav Hrabak

**Affiliations:** ^1^ Department of Microbiology, Biomedical Center, Faculty of Medicine in Pilsen, Charles University, Pilsen, Czechia; ^2^ Institute of Microbiology, Czech Academy of Sciences, BIOCEV, Vestec, Czechia

**Keywords:** mass spectrometry, bacteria, microbiology, MALDI-TOF MS, LC-MS, polysaccharide

## Abstract

**Introduction:**

Over the last two decades, matrix-assisted laser desorption/ionization time-of-flight mass spectrometry (MALDI-TOF MS) has been introduced into the routine diagnostic practice of microbiological laboratories for the rapid taxonomic identification of bacteria and yeasts. However, a method that effectively identifies microbes directly from clinical samples using MALDI-TOF MS has not yet been found. One of the promising targets is microbial polysaccharides, which are abundant structures in bacterial and fungal cells. Their rapid and inexpensive analysis, nevertheless, is complicated. This study focused on detecting microbial polysaccharides, such as lipopolysaccharides, using MALDI-TOF MS and liquid chromatography-tandem mass spectrometry (LC-MS). We developed a method for fingerprinting polysaccharides by acid hydrolysis and enzymatic digestion.

**Methods:**

The mono- and oligosaccharides are then derivatized with a newly designed probe (vanillyl pararosaniline, the HD ligand), enabling efficient ionization without the use of the MALDI matrix. For precise analysis of polysaccharides, the hydroxyl groups can be esterified by formic acid.

**Results:**

The method was validated using several saccharides as well as *Escherichia coli* lipopolysaccharides (O26:B6, O55:B5, and O111:B4). Derivatization using the HD ligand also allows the detection of structures containing amines and phosphate groups in positive ion mode. We optimized the method using crude bacteria (*Escherichia coli*, *Salmonella enterica*, *Shigella dysenteriae*, *Shigella boydii*, *Shigella flexneri*, and *Legionella pneumophila*, *Staphylococcus aureus*) and yeasts *(Candida albicans*, *C. kudriavzevii*, and *C. tropicalis*).

**Discussion:**

This approach opens the possibility of directly detecting microbial polysaccharides from clinical specimens. Laser Desorption/Ionization Time-of-Flight Mass Spectrometry (LDI-TOF MS) using a specific self-ionizable ligand enables direct ionization without the need for an additional matrix, allowing for the particular detection of molecules of interest while suppressing the background signal.

## Highlights

- Expanding the use of MALDI-TOF mass spectrometry in clinical diagnostics
- Novel method for detection of microbial polysaccharides using mass spectrometry
- Ligand (the HD ligand) allowing ionization and analysis of mono- and oligosaccharides

## Introduction

Many innovative applications of matrix-assisted laser desorption/ionization time-of-flight mass spectrometry (MALDI-TOF MS) and liquid chromatography coupled with mass spectrometry (LC-MS) have emerged in clinical diagnostics to detect biomarkers and biomolecules, advancing personalized medicine (Clark et al., 2013; Son et al., 2024).

In diagnostic microbiology, MALDI-TOF MS became a cornerstone in the rapid taxonomic identification of bacteria and fungi (Clark et al., 2013; Kostrzewa, 2018). Similarly, applications for antibiotic resistance determination have also been developed and validated. Among them is the determination of β-lactamase activity, which relies on detecting molecular mass changes of indicator β-lactam antibiotics (e.g., carbapenems) and has already been successfully implemented in routine laboratory practice (Hrabák et al., 2013). Another method allows the detection of polymyxin resistance using analysis of lipid A of lipopolysaccharides and their structural changes (e.g., the addition of phosphoethanolamine or 4-amino-L-arabinose) (Dortet et al., 2020).

As MALDI-TOF MS provides efficient and rapid species identification, there is a key issue of whether the method can be used for epidemiological typing directly from spectra obtained from a whole-cell lysate. To date, no general typing algorithm has been proposed; however, specific peaks representing significant epidemiological markers have been identified in particular species (Kostrzewa, 2018).

Artificial intelligence for spectral analysis has recently been highlighted as a promising tool for predicting antibiotic resistance and supporting epidemiological typing (Weis et al., 2022). While artificial intelligence and machine learning are increasingly applied to large-scale datasets, MALDI-TOF MS remains a fundamentally biochemical technique, designed for the precise detection of molecular weights and patterns of molecular fragmentation. Computational methods can reveal patterns, yet we must always consider the biochemical nature of the signals and understand what is being detected. Current microbiological applications often overlook the precise origin of identified peaks; however, the scientific community should not abandon this goal. With the growing availability of whole-genome sequencing data for microbes, linking spectral features to their genetic determinants will become increasingly relevant and attainable.

On the other hand, while proteins can usually be analyzed by MALDI-TOF MS directly from crude cell extracts or after simple formic acid extraction, the precise identification of other molecules, such as lipopolysaccharides, requires dedicated sample preparation protocols and optimized MALDI-TOF MS ionization techniques.

Despite the commonly used taxonomic applications in routine laboratories, methods for identifying microbes directly from clinical specimens have not yet been successfully developed (Kostrzewa, 2018). Such applications are limited by the low bacterial concentration, which reduces MALDI-TOF MS sensitivity, as well as by the masking of bacterial proteins by highly abundant human proteins present in the sample. These challenges can be addressed by (a) concentrating microbes or microbial proteins in the sample and removing host proteins, (b) detecting other molecules (e.g., lipids or polysaccharides) that can be explicitly extracted from the sample, and (c) using LC-MS.

Identification of microbial polysaccharides in clinical laboratories has a high potential, as these structures can be used for (a) rapid and cheap epidemiological typing (e.g., *Salmonella enterica*, *Shigella* spp., *Escherichia coli*), (b) development of polysaccharide vaccines and testing their efficiency, and (c) direct identification of microbes from clinical specimens. The latter option is currently at the forefront of interest, as microbial saccharides are relatively stable molecules that could be directly detected in clinical specimens (e.g., blood, urine), enabling rapid identification of the causative agent and thus improving therapeutic efficiency (Farrington and Rubenstein, 1991; Viasus et al., 2022; White, 2024).

Since microbial polysaccharides typically consist of more than ten basic units, their molecular weight far exceeds the effective detection range of MALDI-TOF MS or LC-MS for direct analysis. Therefore, the native molecules must be specifically cleaved into smaller units, providing a specific molecular fingerprint. For that purpose, the glycosidic bond is the common target. Hydrolysis of that bond, however, is challenging due to its stability in some biological structures. Several procedures of polysaccharide fingerprinting, including chemical cleavage by strong acids or Fenton’s reaction and enzymatic disruption, have been developed and optimized (Han and Costello, 2013; Amicucci et al., 2020; Urakami and Hinou, 2024).

Even if monosaccharides or oligosaccharides are available, their ionization using conventional mass spectrometry techniques is a complex process. Compared to peptides and proteins, those molecules are generally more complicated to ionize and transfer to the vapor phase (Han and Costello, 2013). Therefore, the typical approach is to derivatize those molecules before mass spectrometry. For that purpose, several methods have been proposed, including the label-assisted laser desorption/ionization approach (Lageveen-Kammeijer et al., 2022).

Here, we present a novel method for enzymatic and acidic hydrolysis of bacterial polysaccharides, their derivatization, and analysis, where the sample is ionized through the addition of a specific ligand, enabling the use of LDI-TOF (Laser Desorption/Ionization Time-of-Flight) MS and LC-MS (Trapped Ion Mobility Spectrometry—TIMS). This approach allows the identification of saccharide fingerprints based on their ion mobility and *m/z* value. Additionally, we proposed the esterification of mono- and oligosaccharides by formic acid, which can aid in their identification.

## Materials and methods

### Reagents

For analysis, all chemicals (_D_-glucose, _D_-glucose-1,2-^13^C_2_, glucose-6-phosphate, _L_-glucose, _D_-fructose, _L_-rhamnose, _L_-fucose, _D_-xylose, lactose, maltose, sucrose, raffinose, 5-(hydroxymethyl)furfural), starch, agarose, lipopolysaccharides (*Escherichia coli* O26:B6, *E. coli* O55:B5, *E. coli* O111:B4), enzymes (diastase, α-amylase, β-amylase, lysozyme, chitinase), and other reagents were purchased from Merck (Merck Life Science, Prague, Czech Republic). The greater quantity of vanillyl-pararosaniline (the HD ligand) was synthesized as 2,3-dichloro-5,6-dicyano-1,4-benzoquinone salt by Ratiochem (Brno, Czech Republic).

### Instrumentation

Samples for LC-MS were injected and subsequently desalted online using reverse-phase microtrap column (Neo Trap Cartridge 5mm, ThermoFischer Scientific, Prague, Czech Republic) and separated on the reverse-phase analytical column (Bruker TEN, Bruker Daltonics, Bremen, Germany) at 40 °C using Bruker nanoElute 2 HPLC system at flow rate 0.5 µL/min in water/acetonitrile gradient (mobile phase [A] water with 0.1% formic acid; mobile phase [B] acetonitrile with 0.1% formic acid; the gradient started at 10% [B] and reached 50% [B] in 35 min). The eluted analytes were directly analyzed by timsTOF Pro 2 mass spectrometer using a CaptiveSpray for ionization (Bruker Daltonics, Bremen, Germany). Measurement was performed in positive ion mode over the *m/z* range 100–1350 in dia-PASEF^®^ mode (0.75 – 1.60 V.s/cm^2^, ramp time 100 ms). Data acquisition and data processing were performed using DataAnalysis 6.1.

LDI-TOF MS was performed using a MALDI Biotyper^®^ Sirius mass spectrometer and rapifleX^®^ MALDI-TOF/TOF system (Bruker Daltonics, Bremen, Germany). The spectra were analyzed using flexAnalysis 4.0 software.

For precise identification of molecular mass, the samples were analyzed using a 15T solariX XR FT-ICR mass spectrometer (Bruker Daltonics). Mass spectral data were collected in positive broadband mode over the *m/z* range 150 – 1500, with 1M data points transient and 0.2 s ion accumulation with two averaged scans per spectrum. Data acquisition and data processing were performed using ftmsControl 2.1.0 and DataAnalysis 5.0.

### Synthesis of vanillyl-pararosaniline reagent (the HD ligand)

Pararosaniline hydrochloride (basic fuchsin) and vanillin were dissolved in methanol to a 100 mmol/L concentration (both reagents). After dissolving, glacial acetic acid was added to a final concentration of 2.5 mol/L and vortexed for 2 minutes. Subsequently, methylpyridine borane dissolved in methanol as a reducing agent was added to the reaction mixture to a final concentration of 10 mmol/L and incubated at 50 °C with shaking for 2 hrs. The reaction was monitored by MALDI-TOF MS and LC-MS using conditions described above.

**Figure 1:**
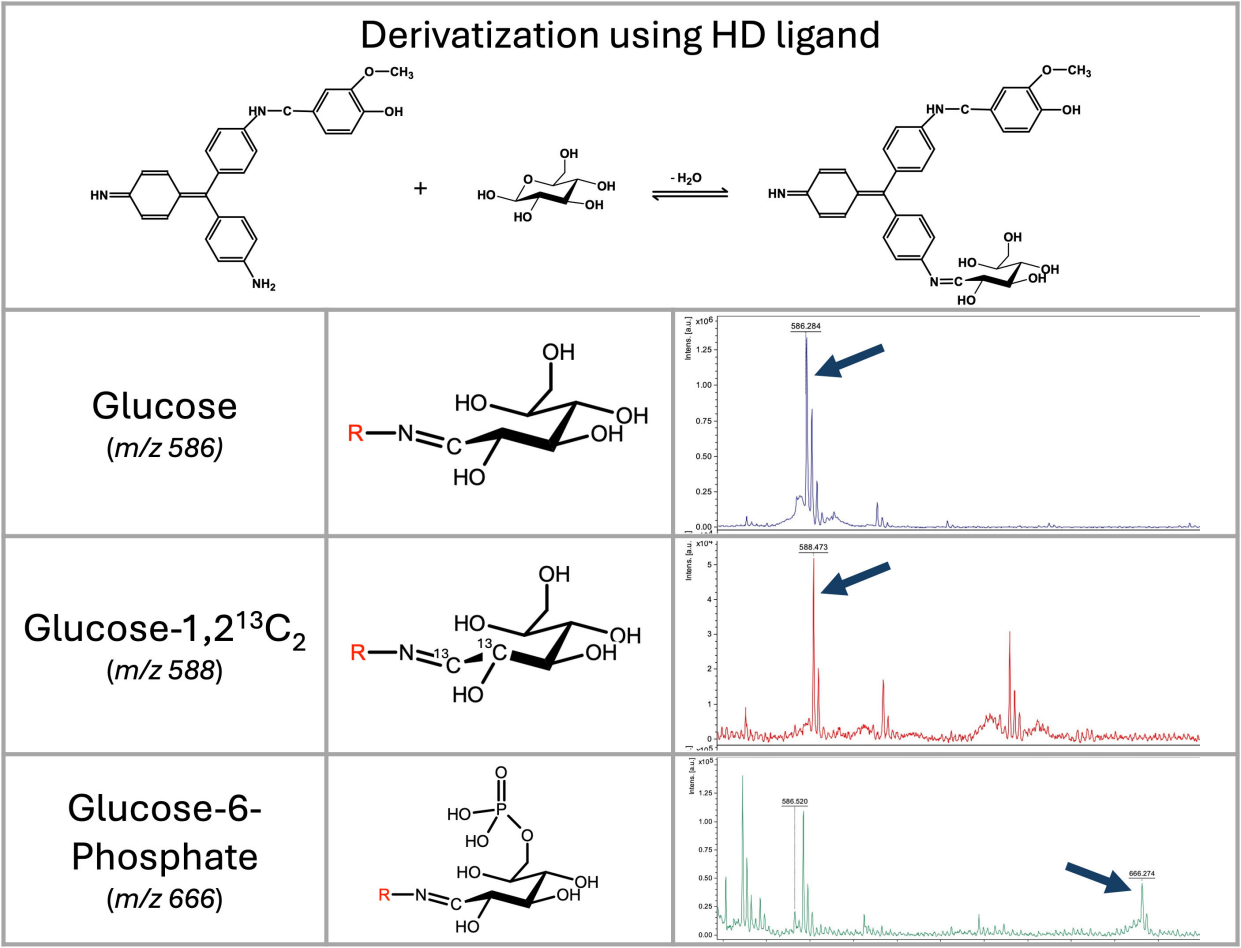
Vanillyl-pararosaniline (HD ligand) mechanism of saccharide’s derivatization, and LDI-TOF MS analysis of selected saccharides. Mass spectra of glucose, _D_-glucose-1,2-^13^C_2_, and glucose-6-phosphate derivatized by the HD ligand measured on a rapifleX mass spectrometer in linear positive ion mode. Derivatized glucose is visible at *m/z* 586; D-glucose-1,2-^13^C is visible as a signal at *m/z* 588; and glucose-6-phosphate as a signal at *m/z* 666.

After incubation, the reaction was diluted by deionized water to a final concentration of methanol of 10% and directly applied on the NGC Quest Plus System (Bio-Rad, Prague, Czech Republic) equipped with a XSelect^®^ CSH C18 OBDTM preparative column (Waters, Gesellschaft m.b.H., Prague, Czech Republic) at a flow rate 0.1 mL/min. After application of the reaction mixture, the column was washed with 50 mL of 5% acetonitrile with 0.1% formic acid. A water/acetonitrile gradient (mobile phase [A] water with 0.1% formic acid; mobile phase [B] acetonitrile with 0.1% formic acid) was used for sample purification, with the gradient starting at 5% [B] and reaching 35% [B] in 60 min. Two mL fractions were collected and measured using LC-MS after dilution with water 1:10. Fractions showing purified vanillyl-pararosaniline (the HD ligand) (**Figure 1**) were dried by a vacuum concentrator.

### Saccharide’s derivatization and analysis

Derivatization was performed in a 1% pyridine buffer, and the pH was adjusted to 4.0 using glacial acetic acid. Saccharides were diluted to a concentration of 5 mmol/L with a reaction buffer. A concentrated HD (250 mmol/L) solution diluted in acetonitrile was added to the reaction mixture to a final concentration of 5 mmol/L. The reaction was incubated at 50 °C for 20 minutes.

For LC-MS, the samples were diluted 1:5 with a pyridine buffer. For LDI-TOF MS, the samples were purified by C18-reversed phase silica gel to avoid the “sweet spot” formation on the MALDI target. Purification was performed in the Eppendorf tube. To 5 mg of C18 reverse phase resin, 10 μL of 30% acetonitrile with 0.1% formic acid was added. After vortexing, 50 μL of the reaction mixture was added, vortexed for 10 s, and centrifuged at 14,000 g. The resin was washed with 1 mL of 5% acetonitrile with 0.1% formic acid. The sample was eluted with 25 μL of 50% acetonitrile with 0.1% formic acid. Two μL of the sample was directly applied to the MALDI target, allowed to dry, and then measured without adding any matrix.

The sensitivity of the method was evaluated using serial dilutions of glucose and lactose (0.01 mmol/L to 100 mmol/L). Data were analyzed using flexAnalysis (for MALDI-TOF MS) and DataAnalysis (for timsTOF) software, respectively. For MALDI-TOF MS spectra, peak detection was performed with the SNAP algorithm (signal-to-noise threshold 6, SNAP average composition: Averagine) in combination with TopHat baseline subtraction. For timsTOF data, peak detection was performed at m/z 586.2 and 748.4 by smoothing the chromatogram (Gaussian algorithm, smoothing width 2, one cycle), followed by baseline subtraction (flatness 0.8).

### Enzymatic digestion of bacterial lipopolysaccharides

Two milligrams of bacterial lipopolysaccharide were dissolved in 100 μL of 25 mM EDTA, pH 7.5, with 1 mg/mL diastase (α-amylase and β-amylase) and 1 mg/mL lysozyme. The reaction was incubated at 50 °C for 30 minutes. After incubation, the reaction was filtered using a Microcon^®^ 10 Centrifugal Filters, 10 kDa NMWL (14,000 g, 30 minutes). To the fraction containing low molecular mass molecules (filtrate collected in the bottom tube), 50 μL of reaction buffer containing 5 mmol/L of the HD ligand was added, and saccharides were derivatized at 50 °C for 20 minutes as described above.

### Acidic digestion of oligo- and lipopolysaccharides

Saccharides and lipopolysaccharides were digested by formic acid and its isotopic variant (^13^C). One milligram of the sample was dissolved in 10 μL of concentrated formic acid or ¹³C-labeled formic acid and incubated at 98 °C for 10 minutes in a thermocycler with a heated lid (104 °C). After incubation, 50 μL of 5% pyridine in water was added. The samples were filtered using Microcon^®^ - 10 Centrifugal Filters, 10 kDa NMWL (14,000 g, 30 minutes). The bottom fraction (50 μL) containing digested saccharides was used for derivatization using the HD ligand and analysis as described above.

### Analysis of lipopolysaccharides from bacterial cells

The bacteria were obtained from the Czech National Collection of Type Cultures (National Institute of Health, Prague, Czech Republic): *Escherichia coli* ATCC25922, *E. coli* O55:B5 CNCTC5874, *E. coli* O111:B4 CNCTC5650, *Salmonella enterica* subsp. *enterica* serovar Enteritidis CNCTC5187, *S. enterica* subsp. *enterica* serovar Montevideo CNCTC6279, *S. enterica* subsp. *arizonae* CNCTC6478, *Shigella dysenteriae* CNCTC5204, *Shigella boydii* CNCTC6338, *Sh. flexneri* serovar 1a I:2,4 CNCTC6370, *Sh. flexneri* CNCTC6378 serovar 4a IV:3,4, and *Staphylococcus aureus* ATCC29213. *Legionella pneumophila* serogroups 1 (sequence type (ST) 1), *Candida albicans*, *Candida kudriavzevii* (formerly *C. krusei*), *Candida tropicalis* were obtained from our collection at the Department of Microbiology, Faculty of Medicine and University Hospital in Pilsen, Czech Republic. All members of the Enterobacterales order were cultivated on Mueller-Hinton agar at 35 °C overnight. *Legionella pneumophila* was cultivated on BCYE agar in an atmosphere with 5% CO2 for 24 hrs. The full loop of the bacteria was resuspended in 1 mL of 96% ethanol, centrifuged, and then allowed to dry at 98 °C for 5 minutes. Twenty microliters of concentrated formic acid were added to the pellet and incubated at 98 °C with shaking (1200 rpm) for 15 minutes. After incubation, 100 μL of 5% pyridine was added to adjust the pH to 3.0. The mixtures were then filtered using an Microcon^®^ - 10 Centrifugal Filters, 10 kDa NMWL (14,000 g, 30 minutes), and the bottom filtrate (50 μL) was used for derivatization using the HD ligand as described above. For microbes, further purification of derivatized saccharides is not required. Therefore, one microliter of the reaction mixture was directly applied to the MALDI target. As a control (negative control, control of digestion and derivatization), 1 mg of glucose and lactose has been used. For LDI-TOF MS, peak detection was performed with the SNAP algorithm (signal-to-noise threshold 6, SNAP average composition: Averagine) in combination with TopHat baseline subtraction.

## Results

### Synthesis of the HD ligand

Initially, we tested the derivatization of saccharides using pararosaniline. Despite the very efficient binding of aldoses to pararosaniline, the molecule’s disadvantage was the presence of two efficient binding sites (NH_2_) and the requirement for a matrix for MALDI-TOF MS analysis. Therefore, we had tested several molecules containing an aldehyde residue to block one of those amines. By using vanillin, we obtained a molecule with excellent ionization ability that does not require the use of any matrix in MALDI-TOF MS, a laser desorption/ionization time-of-flight (LDI-TOF) MS approach. It was, however, necessary to enhance the stability of the molecule by reductive amination of Schiff’s base (**Supplementary Figure S1**). Since a conjugated system is essential for efficient molecule ionization, we have tested an appropriate reducing agent (e.g., sodium cyanoborohydride, borane pyridine complex, 2-methylpyridine borane complex). Finally, we have chosen a 2-methylpyridine borane complex because the reaction can proceed in a single step. It is also important to note that high concentrations of reducing agents, as recommended for conventional reactions, lead to the formation of leuco base of pararosaniline, a colorless reduced form. That molecule has very low ionization ability and requires a classical matrix-based MALDI-TOF MS setup (data not shown).

Synthesis of the HD ligand was performed as outlined in the Materials and Methods section. During the synthesis, all amines of pararosaniline reacted with the aldehyde group of vanillin, yielding mono-, di-, and tri-vanillin derivatives (m/z 424, 560, and 696). LC-MS analysis revealed that the mono- and di-vanillin variants were present in equal proportions, indicating high efficiency of aldehyde binding. In contrast, the concentration of the tri-vanillin variant was below 10%. In the absence of the reducing agent (methylpyridine borane), the observed molecular masses of all variants (m/z 422, 556, 691) indicated the formation of Schiff bases.

The purity of the HD ligand synthesized in our laboratory was determined using LC-MS, and the structure was verified by solariX magnetic resonance mass spectrometry (Bruker Daltonics, Bremen, Germany) (**Supplementary Figure S2**). The quality of the commercially synthesized HD ligand, available as a 2,3-dichloro-5,6-dicyano-1,4-benzoquinone salt (DDQ), was determined by nuclear magnetic resonance (NMR) spectroscopy (**Supplementary Figure S3**). Both variants showed equal results in the following experiments.

### Derivatization of mono- and disaccharides

The reaction was initially optimized using glucose and lactose as substrates. The best results were obtained at a low pH (3-4) and in a buffer that did not contain chlorine ions. Therefore, we selected a pyridine buffer of pH 3.0 for further experiments. Using a MALDI-TOF mass spectrometer, the HD ligand exhibited excellent ionization ability for detecting relevant signals of the conjugate with saccharides (**Figure 1**) without the need for a matrix (LDI-TOF MS approach).

Ionization was performed in a positive ion mode with the analytes detected as [M+H]^+^ ions. All the saccharides tested provide a signal with a mass-to-charge ratio that can be calculated by the following formula (see **Table 1**):

**Table 1:** LDI-TOF MS and LC-MS analysis of mono-, disaccharides, and 5-(hydroxymethyl)furfural showing *m/z* and ion mobility of derivatized molecules.

Saccharide	*m/z*	Ion mobility [V·s/cm^2^]
_D_-glucose	586	1.242
_L_-glucose	586	1.242
_D_-glucose-1,2-^13^C_2_	588	1.242
glucose-6-phosphate	666	1.219
_L_-rhamnose	570	1.224
_L_-fucose	570	1.228
_D_-xylose	556	1.210
lactose	748	1.503
maltose	748	1.490
5-(hydroxymethyl)furfural	532	1.099


$$m/z={\left[{\text{M}}_{\text{HD ligand}}+{\text{M}}_{\text{Saccharide}}-{\text{M}}_{\text{H}2\text{O}}+\text{H}\right]}^{+}$$

Where the M_HD_ ligand is 423.195 (monoisotopic mass), M_H2O_ represents the loss of water (-18.015) during the formation of a Schiff base.

As demonstrated by glucose-6-phosphate, derivatization with the HD ligand enables the detection of molecules with strongly negatively charged groups, such as phosphates, in a positive ion mode as well (**Figure 1**).

A purification step using C18 resin can be performed using the resin itself (as described in the methodology) or with ZipTips^®^, which enhances the ionization ability in LDI-TOF MS analysis of mono- and oligosaccharides. This step removes unbound sugars that otherwise form a “sweet spot” and decreases the method’s sensitivity.

**Figure 2:**
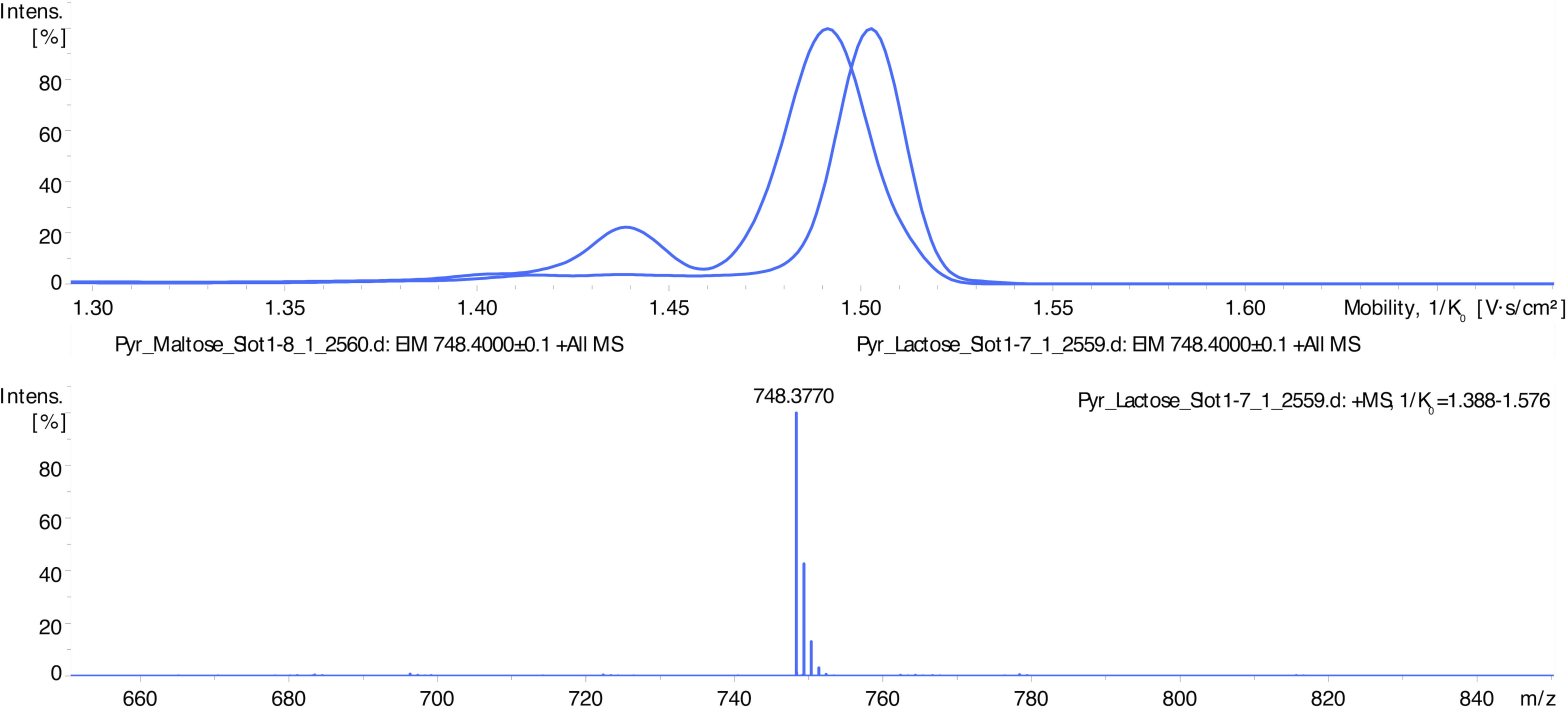
LC-MS spectra showing differentiation of disaccharides’ isoforms by ion mobility determination. Disaccharides maltose and lactose appear as signals at *m/z* 612 (HD ligand with the lost vanillin molecule) and *m/z* 748 (intact HD ligand). Maltose complex (*m/z* 748) shows an ion mobility of 1.490 V.s/cm^2^ and lactose 1.503 V.s/cm^2^.

In LC-MS analysis, derivatized mono- and di-saccharides have also been detected at the expected mass-to-charge ratio (**Table 1**, **Figure 2**). The ion mobility of the saccharides allows their further analysis and identification (e.g., discrimination between a disaccharide, lactose, and maltose – see **Figure 2**). Unfortunately, we were unable to distinguish between D- and L-glucose using our instrument.

For both methods (LDI-TOF MS and LC-MS), the sensitivity was determined to be 0.1 mmol/L, corresponding to the lowest analyte concentration that consistently produced a detectable signal above the baseline noise.

### Acidic digestion and Fischer-Speier esterification

**Figure 3:**
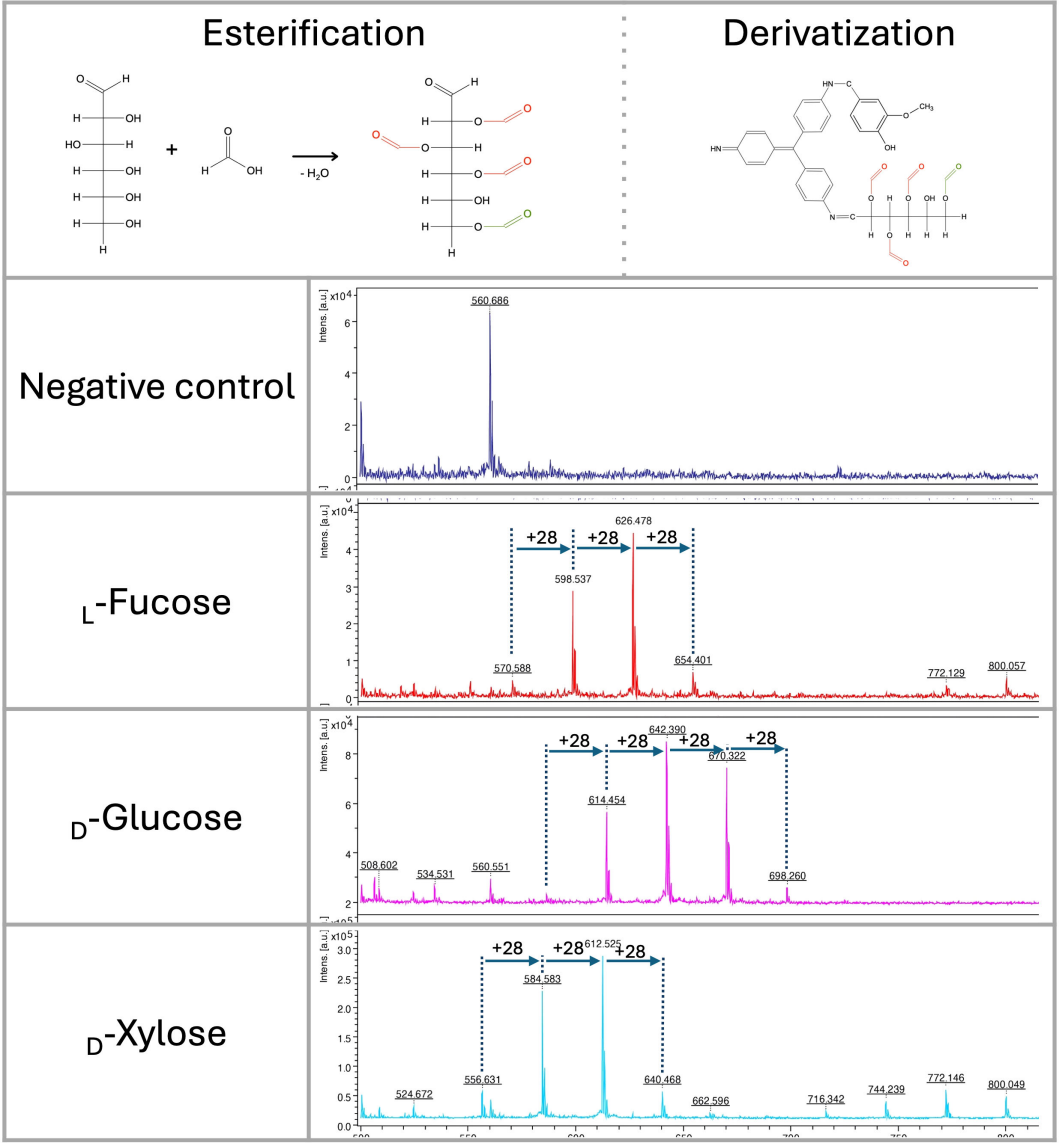
Fischer-Speier esterification of glucose using concentrated formic acid at 98 °C and its derivatization by the HD ligand. Esterification of the hydroxyl group at position 6 (green color) was immediately detected. Esterification of other positions was identified in different ratios. LDI-TOF MS spectra of monosaccharides (negative control, _L_-fucose, _D_-glucose, _D_-xylose) esterified by concentrated formic acid (+*m/z* 28).

Using acidic digestion of oligosaccharides, we identified peaks that did not correspond with simple derivatized mono- and oligosaccharides. Using monosaccharides (_D_-glucose, _L_-fucose, _D_-xylose), formic acid, and isotopic formic acid-^13^C, the peaks corresponding to esters formed in hydroxyl groups of saccharides can be detected. In this reaction (Fischer-Speier esterification), the peaks are shifted by 28 g/mol, corresponding to formic acid-derived esters (**Figure 3**).

In glucose, the hydroxyl group at position 6 is almost completely esterified (*m/z* 614) with a very low signal of derivatized native glucose (*m/z* 586) (**Figure 3**). All hydroxyl groups except one are also esterified with a different ratio, as indicated by signals at *m/z* 642, 670, and 698. Similar results were obtained with _D_-xylose and _L_-fucose, showing no signal with non-esterified derivatized saccharides (*m/z* 556 and 570). As observed in glucose, variants of all esterified hydroxyl groups except one could be detected in LDI-TOF spectra (**Figure 3**). Based on those characteristics, we hypothesize that only one hydroxyl group of vicinal diols can be esterified efficiently. The esterification mechanism on _D_-glucose, _D_-xylose, _L_-fucose, and lactose was confirmed by precise molecular mass determination (<1 ppm) using solariX XR FT-ICR mass spectrometer (**Supplementary Figure S4**).

**Figure 4:**
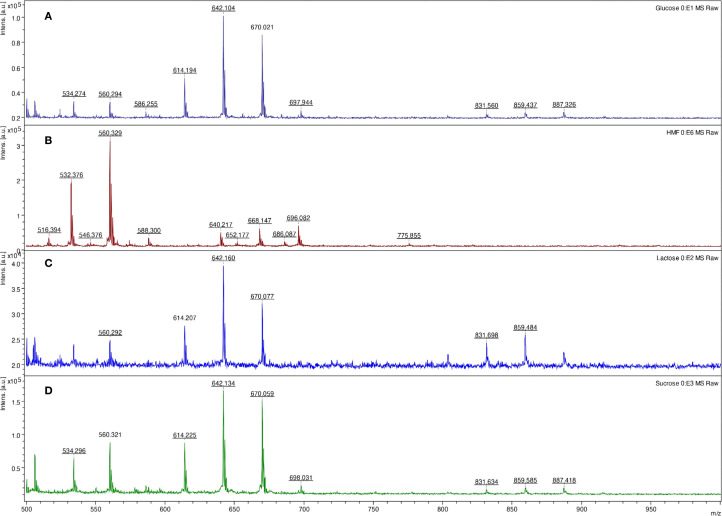
LDI-TOF MS spectra of glucose **(A)**, 5-(hydroxymethyl)furfural **(B)**, lactose **(C)**, and sucrose **(D)** after digestion (lactose, sucrose) and esterification using concentrated formic acid at 98 °C for 10 minutes and derivatization using the HD ligand.

**Figure 5:**
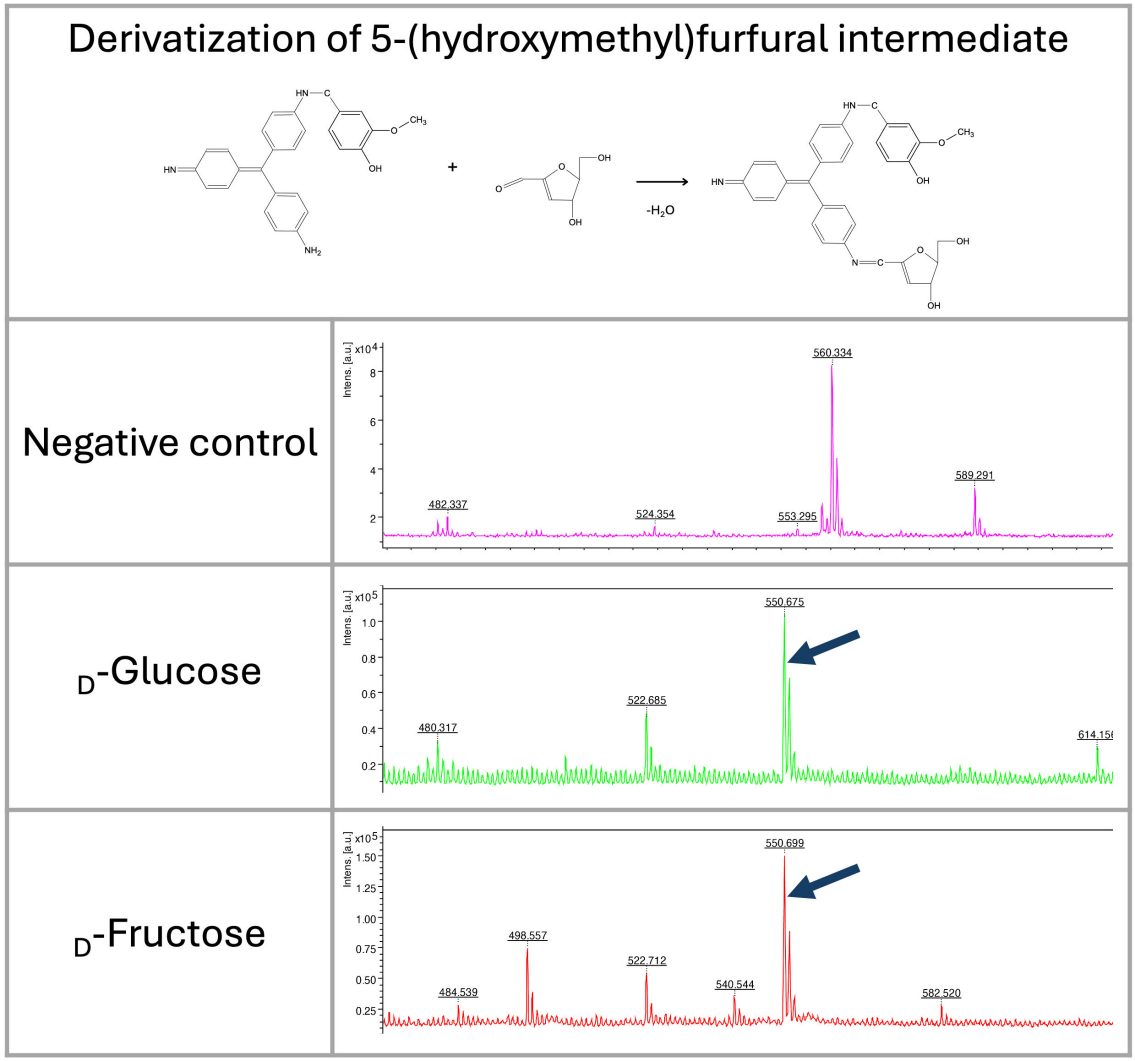
LDI-TOF MS spectra show the formation of 5-(hydroxymethyl)furfural intermediate from fructose and glucose after heating at 98 °C for 30 minutes (*m/z* 550). The molecule was derivatized by HD ligand and measured using LDI-TOF.

As hexoses in acidic conditions and high temperatures can be dehydrated to form 5-(hydroxymethyl)furfural, we also focused on identifying this molecule in the reaction. The 5-(hydroxymethyl)furfural can also be recognized as a signal at *m/z* 532 in acidic conditions. In glucose and fructose, the 5-(hydroxymethyl)furfural intermediate formed during saccharide dehydration (Kognou et al., 2022) was identified in the spectra at *m/z* 550 (**Figures 4**, **5**).

### Analysis of bacterial polysaccharides by acidic digestion

**Figure 6:**
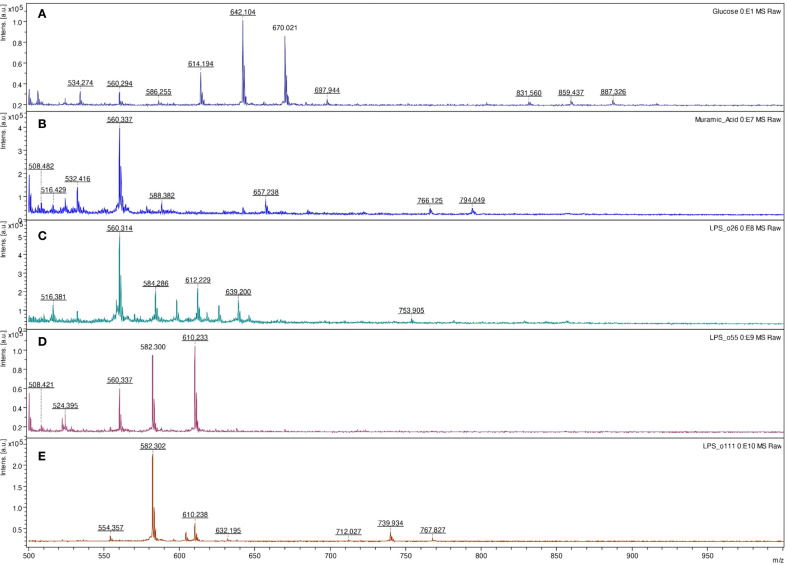
LDI-TOF MS spectra of glucose **(A)**, muramic acid **(B)**, *Escherichia coli* O26:B6 lipopolysaccharide **(C)**, *E. coli* O55:B5 lipopolysaccharide **(D)**, and *E. coli* O111:B4 lipopolysaccharide **(E)** after digestion and esterification using concentrated formic acid at 98 °C for 10 minutes and derivatization using the HD ligand.

Initially, formic acid was tested for non-specific fingerprinting of polysaccharides. As demonstrated in **Figure 4**, concentrated hot formic acid (98 °C) can efficiently hydrolyze glycosidic bonds. The same results were obtained in raffinose and starch (data not shown). The method also allows for the detection of common saccharides in the bacterial cell wall, specifically muramic acid, which contains an amine and carboxyl group (*m/z* 657) (**Figure 6**).

**Figure 7:**
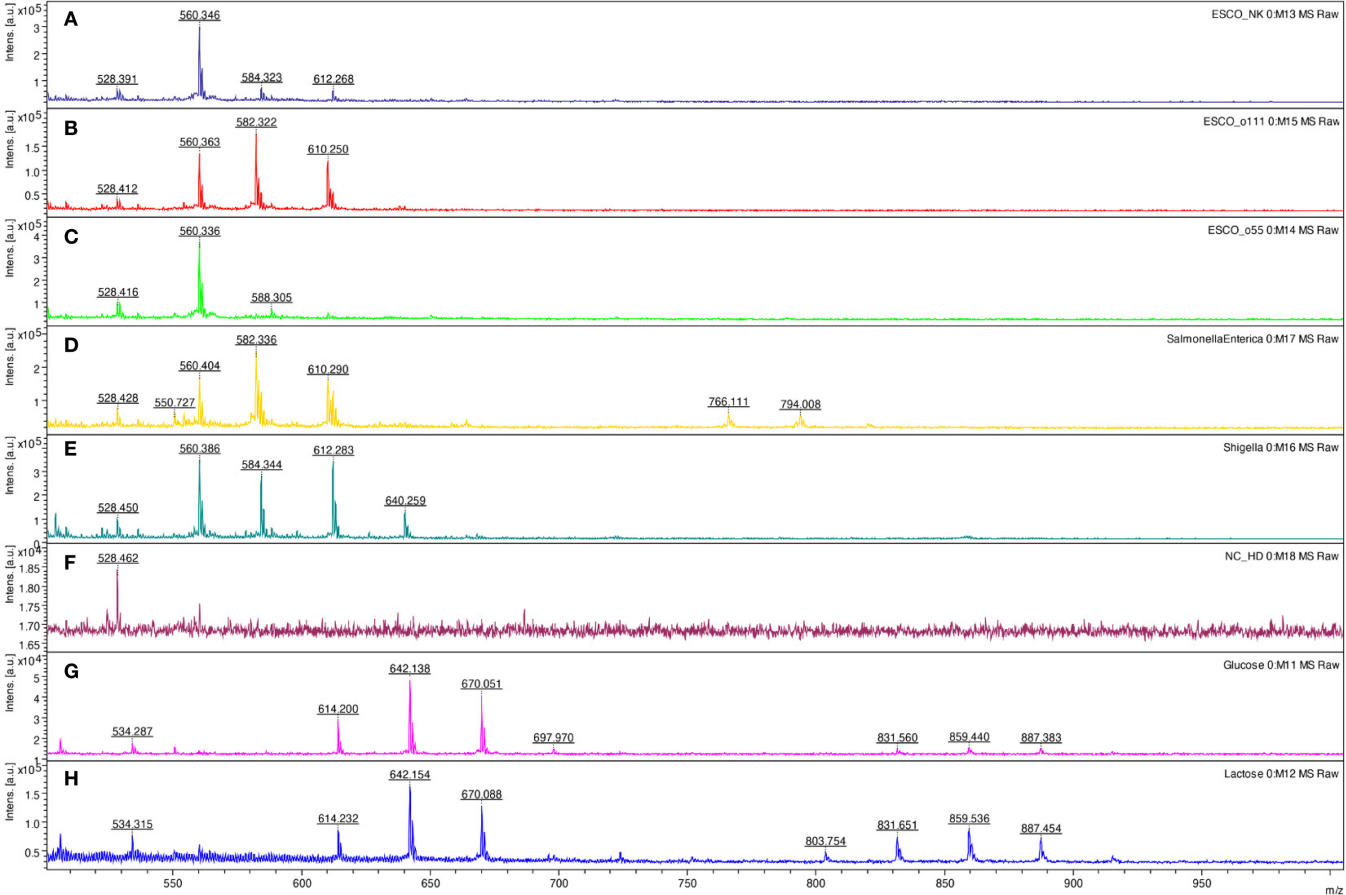
LDI-TOF MS spectra of *Escherichia coli* ATCC25922 **(A)**, *E. coli* O111:B4 CNCTC5650 **(B)**, *E. coli* O55:B5 CNCTC5874 **(C)**, *Salmonella enterica* subsp. *enterica* serovar Enteritidis CNCTC5187 **(D)**, *Shigella dysenteriae* CNCTC5204 **(E)**, negative control – HD ligand only **(F)**, _D_-glucose **(G)**, and lactose **(H)** after digestion and esterification using concentrated formic acid at 98 °C for 10 minutes and derivatization using the HD ligand.

Based on those mono- and oligosaccharide results, the method was tested for fingerprinting bacterial lipopolysaccharides. Comparing three different bacterial polysaccharides of *Escherichia coli*, the results demonstrated different patterns. The same results were obtained for purified lipopolysaccharides of *E. coli* O26:B6, *E. coli* O55:B5, and *E. coli* O111:B4, as well as crude bacteria (**Figure 7**). Similarly, all tested bacteria, including *Salmonella* spp., *Shigella* spp., *L. pneumophila*, and *S. aureus* provided specific patterns showing that the method can be used for bacterial typing (**Table 2**). Glucose and lactose were included as controls in each experiment to assess the efficiency of digestion and derivatization. In contrast, when using the HD ligand alone or non-digested bacterial samples, no significant peaks above the noise level were observed (**Figure 7**).

**Table 2:** Peak list obtained by MALDI-TOF MS analysis for the tested bacteria and yeasts.

Species	Detected peaks (*m/z*)
_D_-glucose	586, 614, 642, 670, 697, 831, 859, 887
*Escherichia coli* ATCC25922	584, 588, 612, 640
*Escherichia coli* o55	588, 610
*Escherichia coli* o111	582, 610, 640
*Salmonella enterica* subsp. *enterica* serovar Enteritidis	582, 610, 612, 766, 794
*Salmonella enterica* subsp. *enterica* serovar Montevideo	582, 584, 608, 610, 612, 766, 794
*Salmonella enterica* subsp. *arizonae*	582, 632, 662, 766, 794
*Shigella dysenteriae*	584, 612, 632, 859
*Shigella boydii*	584, 600, 632
*Shigella flexneri* serovar 1a I:2,4	584, 612, 646, 662, 736
*Legionella pneumophila*	584, 600, 632, 662
*Staphylococcus aureus*	586, 657, 684, 713, 763, 799, 852, 906
*Candida albicans*	578, 586, 632, 721, 856
*Candida kudriavzevii* (formerly *C. krusei*)	578, 586, 632, 651, 658, 721, 743, 754, 856
*Candida tropicalis*	578, 586, 588, 618, 632, 722, 743, 754, 856

### Analysis of polysaccharides by enzymatic digestion

To analyze polysaccharides and lipopolysaccharides, α-amylase and β-amylase were tested using starch as a positive control. In starch, mono—(*m/z* 586), di—(*m/z* 748), and trisaccharide (*m/z* 910) can be observed using both methods (LDI-TOF MS and LC-MS). We also detected distinct lipopolysaccharide profiles (*E. coli* O26:B6, *E. coli* O55:B5, *E. coli* O111:B4) similar to acidic digestion.

## Discussion

We describe here a novel method for derivatizing mono- and oligosaccharides, which can be used for the identification and analysis of these molecules, not only those of microbial origin. Importantly, this approach complements the currently established MALDI-TOF MS–based detection of bacterial and fungal proteins used for taxonomic identification. It does not aim to replace classical protein-based identification, since polysaccharide analysis generally provides different types of information. With only rare exceptions, polysaccharides cannot reveal information on antimicrobial resistance or virulence, which are predominantly determined by proteins.

Initially, we focused on developing a technique to identify bacterial cell-wall polysaccharides (i.e., lipopolysaccharides). We tested several derivatizing agents, as saccharides cannot be easily ionized compared to peptides/proteins by MALDI-TOF mass spectrometry. Inspired by a detection of lactose fermentation using a pararosaniline (basic fuchsin) in diagnostic bacteriology (e.g., Endo agar), we tested that molecule to form a Shiff base between the aldehyde group of reducing sugars and the amine of the fuchsin. This complex could be ionized by MALDI-TOF MS using a standard matrix (e.g., 2,5-dihydroxybenzoic acid). The disadvantage of this process is the presence of two efficient amine residues in the molecule, which are responsible for the polyvalent binding of the tested saccharides. However, the ability to modify saccharides with fuchsin and subsequent ionization showed excellent results. Therefore, we decided to focus our research further on modifying this molecule.

After modifying the pararosaniline (basic fuchsin) by adding vanillin to one of the amines, which was designated the HD ligand, the ionization of the complex with saccharides was allowed without using the matrix (LDI-TOF MS approach). The complex can also be analyzed using LC-MS with separation on a C18 reverse-phase column. As demonstrated in the results (**Figure 1**), some isomeric saccharides can also be distinguished by their ion mobility using trapped ion mobility technology.

The HD ligand enables the derivatization and analysis of saccharides with various substituents, including phosphates, in positive ion mode. This makes the method universal to detect microbial oligo- and polysaccharides of different origins. However, it is necessary to use optimal reaction conditions for derivatization, including a pH range of 3 to 5. We also found that a high concentration of chlorine ions inhibits the formation of a Schiff base (data not shown). Among the buffers tested, pyridine in concentrations ranging from 1% to 10% with a pH adjusted by formic or acetic acid provided the highest binding efficiency.

Although we expected the pararosaniline-based system also to enable the detection of ketones (ketoses), we were unable to find conditions that allowed for efficient and stable binding of the HD ligand. This may be because the HD ligand requires specific binding conditions or is unstable upon ionization during mass spectrometry. On the other hand, however, ketoses are not common structures in bacterial cells. For analysis, they can be modified to furfurals in acidic conditions that are also analyzable by our system as well (see **Figure 5**).

When different options for the hydrolysis of the glycosidic bond of polysaccharides were tested, we found that using formic acid, spectra containing many ions of different m/z values with regular repetitions (+28) were detected. Through detailed analysis, including the reaction in ^13^C-formic acid, we identified the signals as Fischer-Speier esters formed in the hydroxyl groups of saccharide molecules. This behavior can be further used to identify and analyze saccharides more accurately. For future experiments, the relative intensity of signals representing esterified hydroxyl groups can be further verified to determine their position in the molecule. We also tested acetic acid for esterification. However, its efficiency was significantly lower compared to formic acid. In glucose, an acetic acid-derived ester was formed at position six only (data not shown). Those findings were crucial for further microbial polysaccharide experiments to understand the reaction.

An essential step in analyzing microbial polysaccharides, which typically have a very high molecular weight, is to digest the molecules specifically. Similarly to derivatization methods, we tested various possibilities, including specific lipopolysaccharide isolation methods, such as the Bligh-Dyer solution and its modifications (data not shown); however, the yield was not beneficial compared to the simple *in-situ* polysaccharide digestion. Interestingly, common amylases (α and β) can efficiently digest bacterial polysaccharides to mono-, di-, and trisaccharides. Peptidoglycan-specific enzymes (e.g., lysozyme) can also be used for specific analysis. A similar approach can be optimized to analyze other microbial polysaccharides (e.g., galactomannan and glucan in molds and yeasts) that are clinically relevant for the rapid diagnosis of invasive fungal infections or the detection of resistance to antifungal drugs.

**Figure 8:**
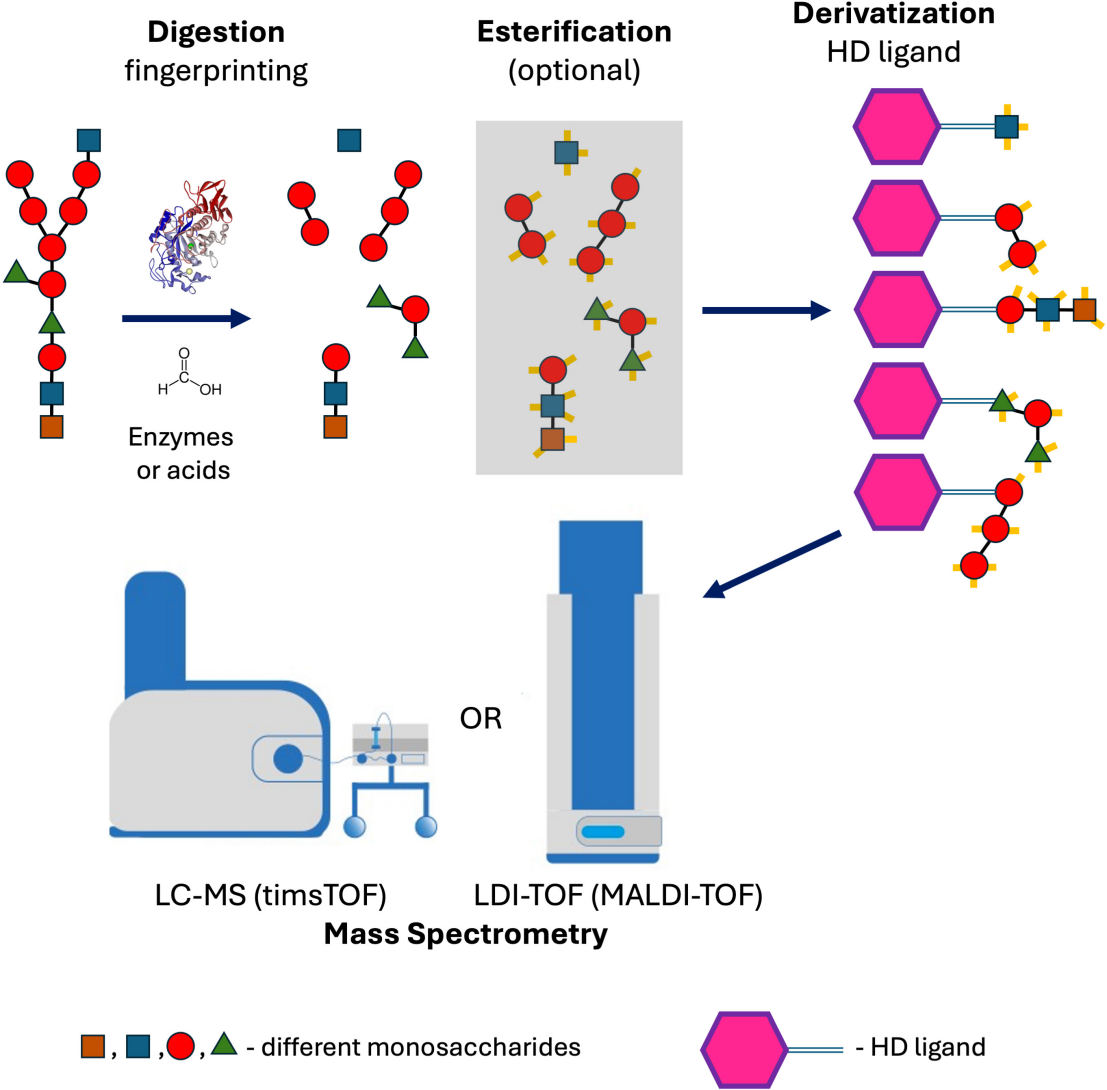
Method workflow showing the analysis of polysaccharides by mass spectrometry, following digestion with formic acid or enzymes and subsequent derivatization with a self-ionizable the HD ligand.

Finally, we selected a straightforward method that does not require any specific extraction of cell-wall polysaccharides: incubating microbes in concentrated formic acid at 98 °C (**Figure 8**). This procedure blocks reactive amines in the crude microbes, and the polysaccharides can be simultaneously digested into mono and oligosaccharides in a one-step process without previous specific extraction of the cell walls, which is usually laborious. That approach is a typical example of applying Ockham’s razor and can be easily used in routine diagnostic laboratories.

In all applications, however, it is crucial to filter the reaction mixture before the derivatization of saccharides to remove undigested polysaccharides (> 3–10 kDa). Without this step, the spectra show an insufficient noise-to-signal ratio.

## Conclusions

The method described here enables the analysis of microbial polysaccharides not only for epidemiological typing and polysaccharide vaccine development, but also opens up the possibility of directly detecting these structures in clinical specimens. Moreover, the HD ligand provides a versatile tool for derivatizing saccharides and related aldehyde-containing molecules (e.g., furfurals), thereby facilitating their analysis by mass spectrometry (LDI-TOF MS, LC-MS). An additional advantage is its applicability in LDI-TOF MS analysis, which eliminates the need for a matrix, allowing for the selective ionization of target molecules while suppressing signals from irrelevant ones.

## Data Availability

The original contributions presented in the study are included in the article/**Supplementary Material**. Further inquiries can be directed to the corresponding author.

## Supplementary material

Supplementary Figure 1 Principle of low-scale preparation of Vanillyl-Pararosaniline (HD) ligand using 2-methylpyridine borane complex as a reduction agent and purification by HPLC system.

Supplementary Figure 2 Confirmation of Vanillyl-Pararosaniline (HD) ligand structure using 15T solariX XR FT-ICR mass spectrometer (Bruker Daltonics).

Supplementary Figure 3 Quality control protocol (NMR spectra) of commercially prepared Vanillyl-Pararosaniline (HD) ligand.

Supplementary Figure 4 Confirmation of esterified _D_-glucose, _D_-xylose, _L_-fucose, and lactose using 15T solariX XR FT-ICR mass spectrometer (Bruker Daltonics).
