# Supplementary figures and images for: Mass spectrometric profiling of microbial polysaccharides using laser desorption/ionization – time-of-flight (LDI-TOF) and liquid chromatography-mass spectrometry (LC-MS): a novel method for structural fingerprinting and derivatization
Source: Front Cell Infect Microbiol. 2025 Oct 3;15:1658802. doi: 10.3389/fcimb.2025.1658802 (PMC12531147; doi:10.3389/fcimb.2025.1658802)

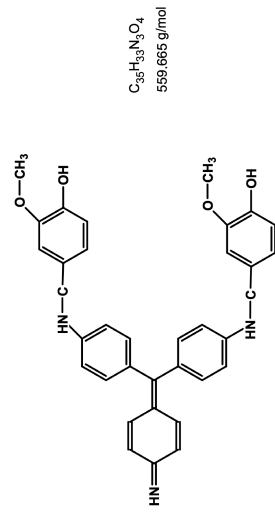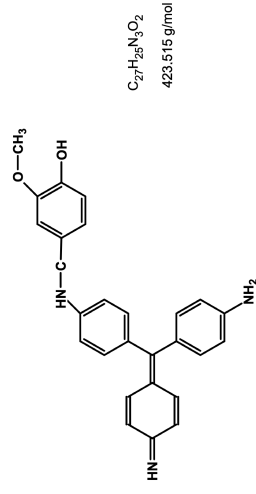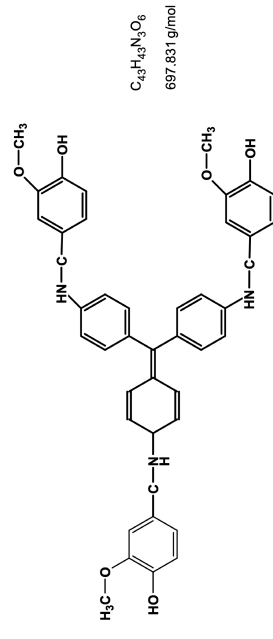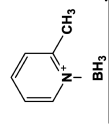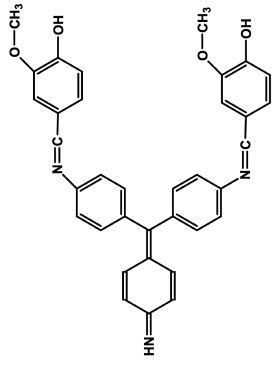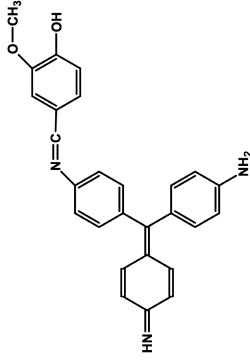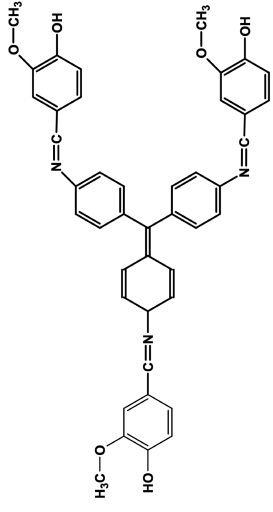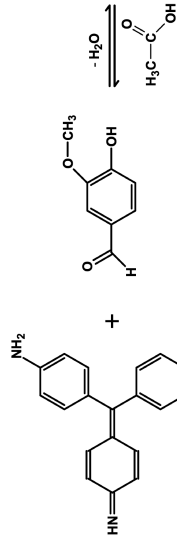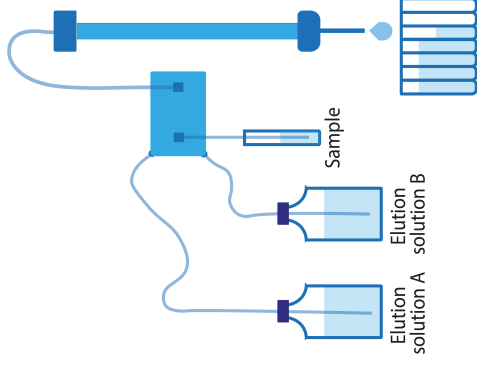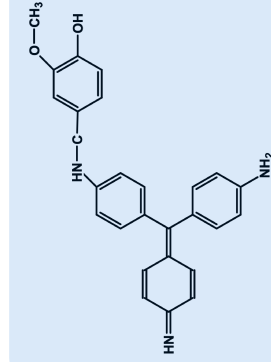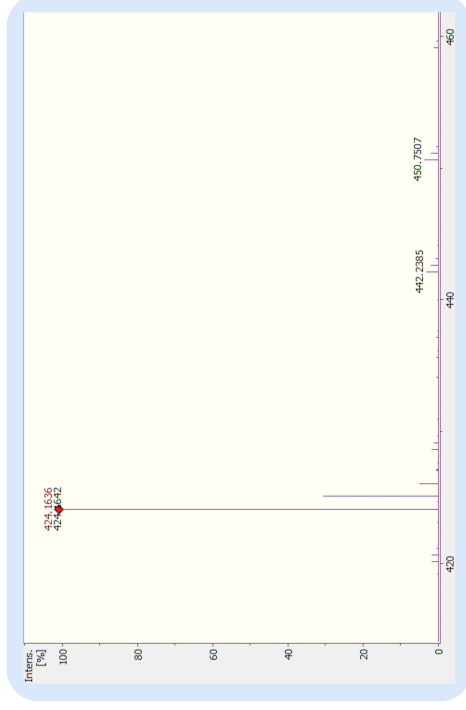

Supplement: Supplementary Figure 1 — Principle of low-scale preparation of Vanillyl-Pararosaniline (HD) ligand using 2-methylpyridine borane complex as a reduction agent and purification by HPLC system. [file DataSheet1.pdf]

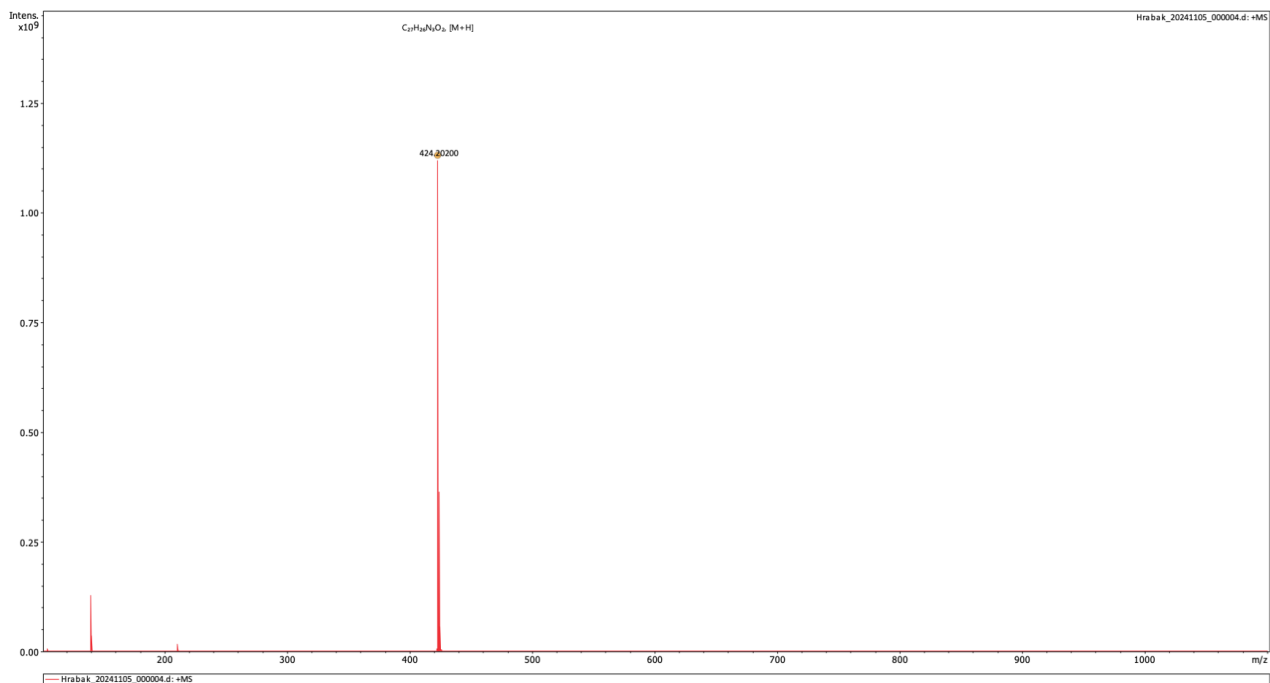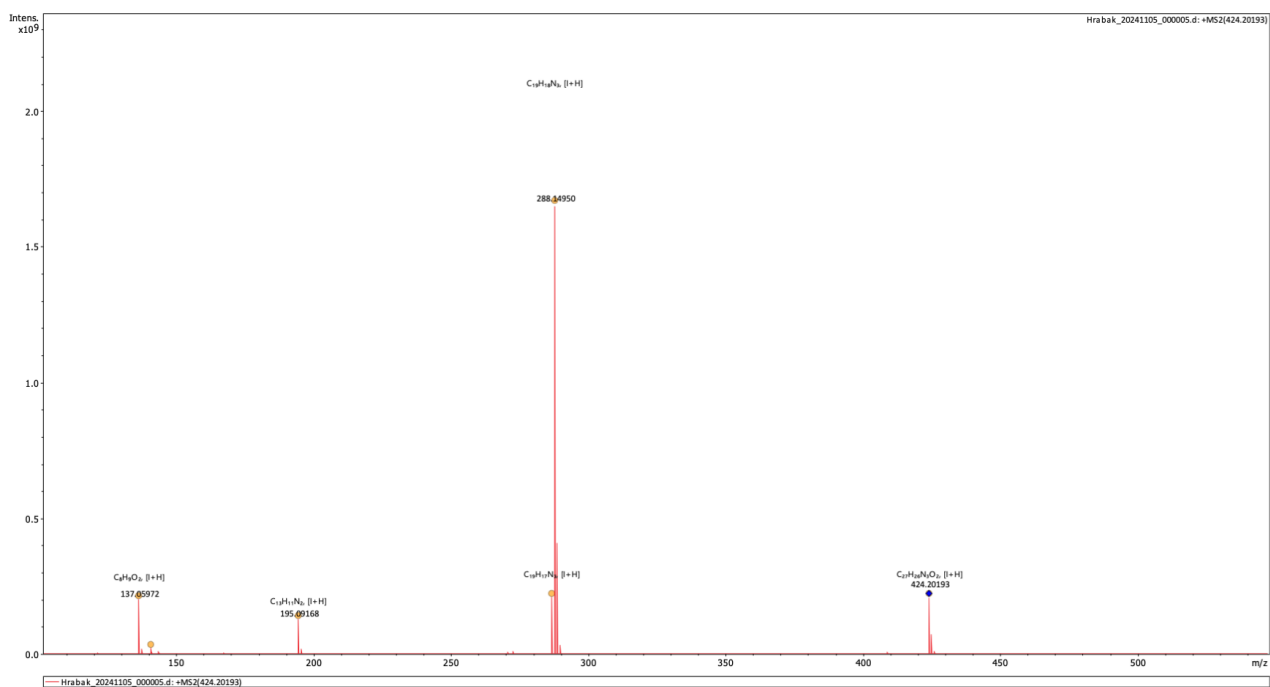

Supplement: Supplementary Figure 2 — Confirmation of Vanillyl-Pararosaniline (HD) ligand structure using 15T solariX XR FT-ICR mass spectrometer (Bruker Daltonics). [file DataSheet2.pdf]

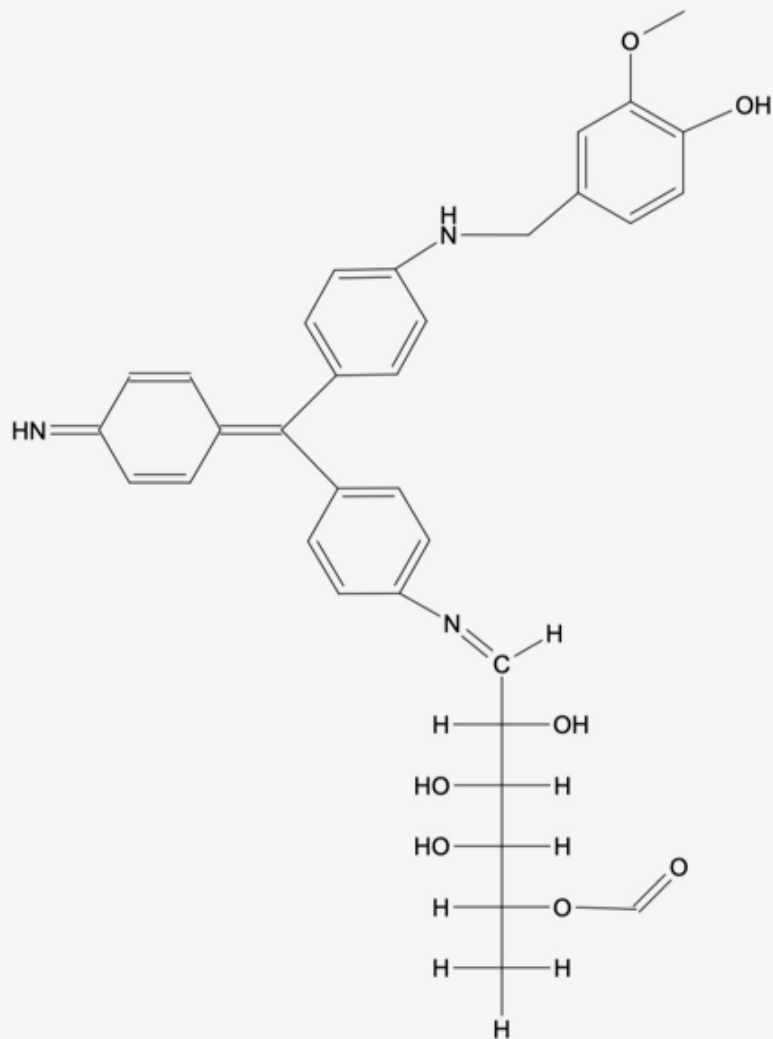

$C_{34}H_{35}N_3O_7$   
597,667 u  
fucose

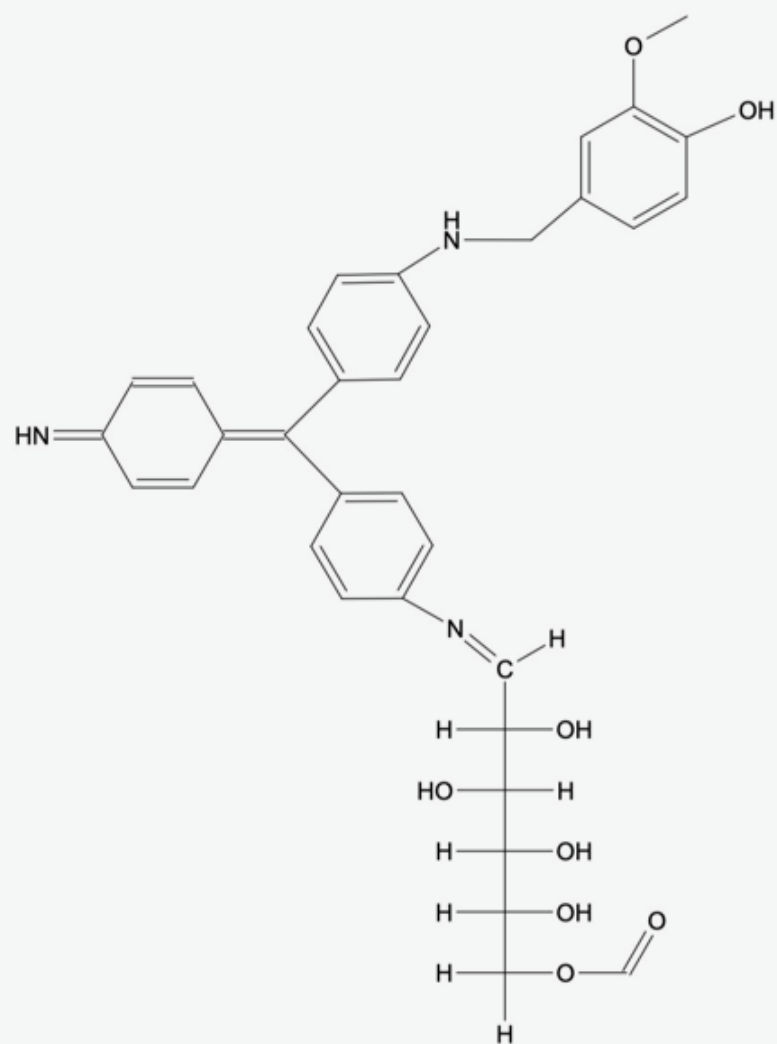

$C_{34}H_{35}N_3O_8$   
613,666 u  
glucose

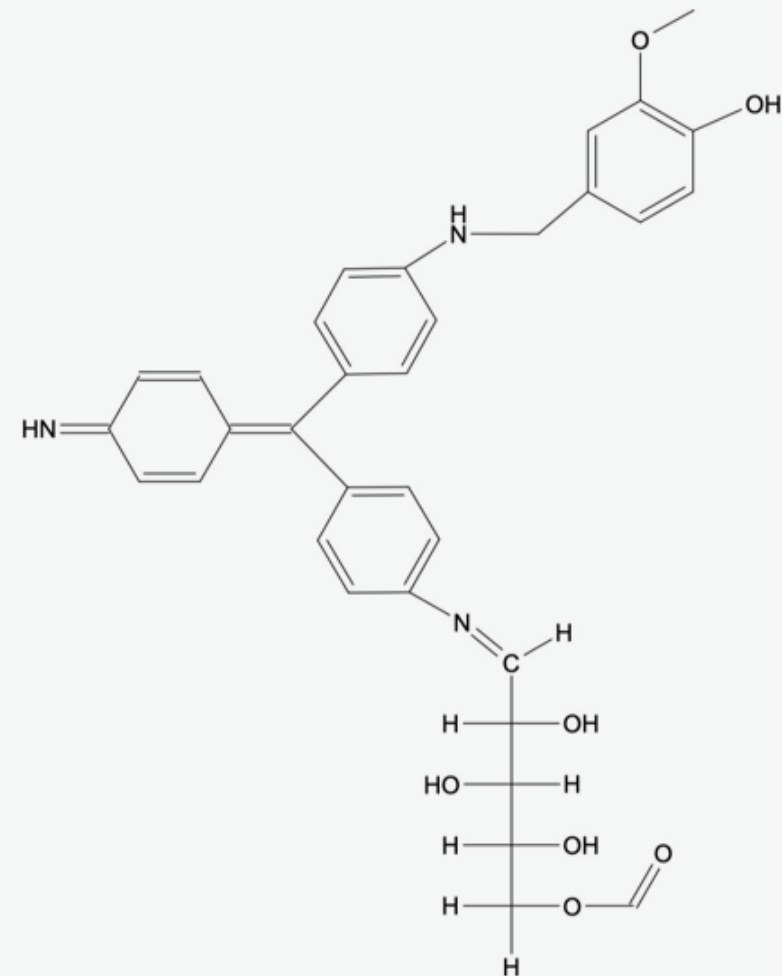

$C_{33}H_{33}N_3O_7$   
583,64 u  
xylose

# Fucose

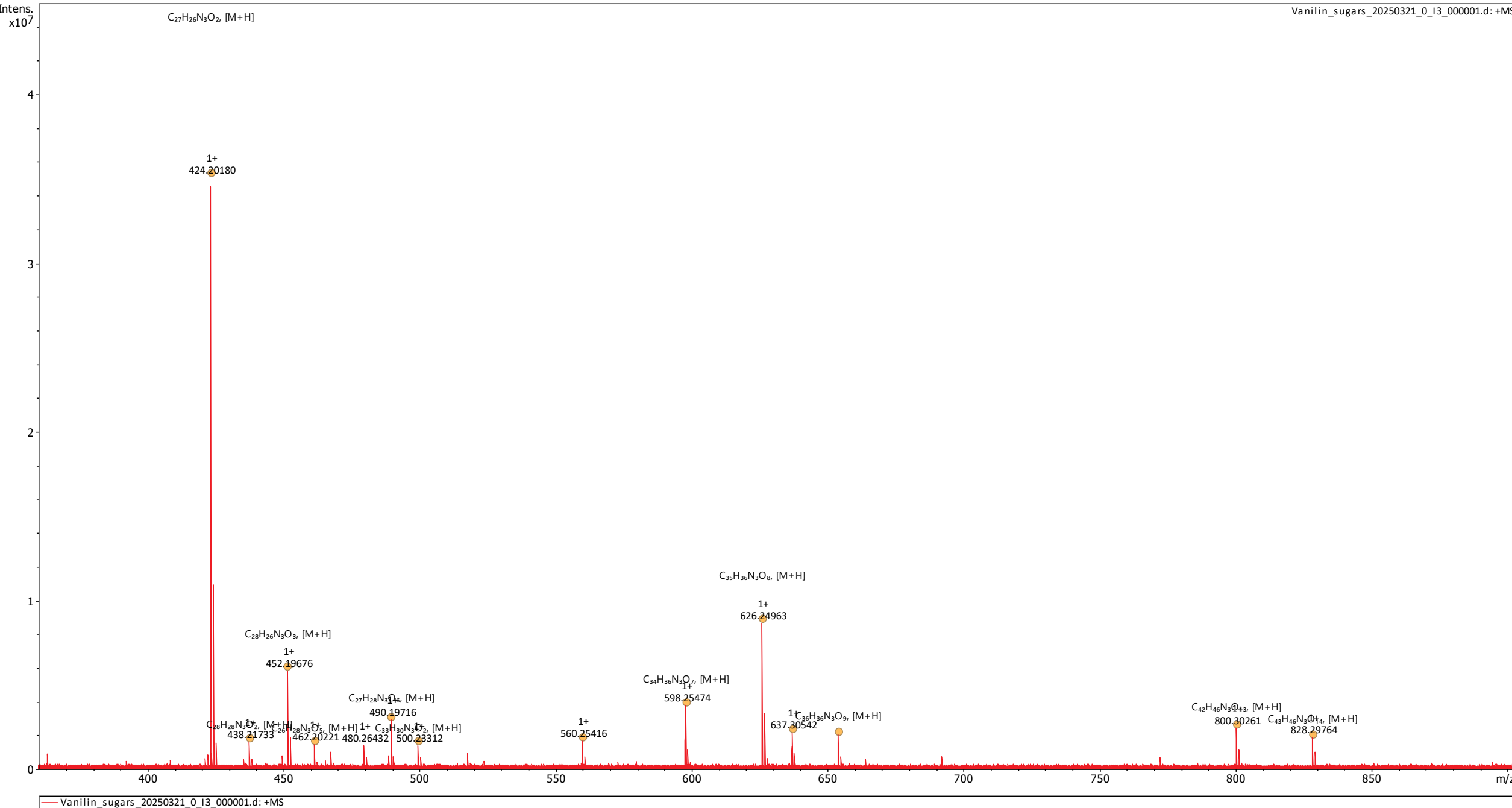

# Glucose

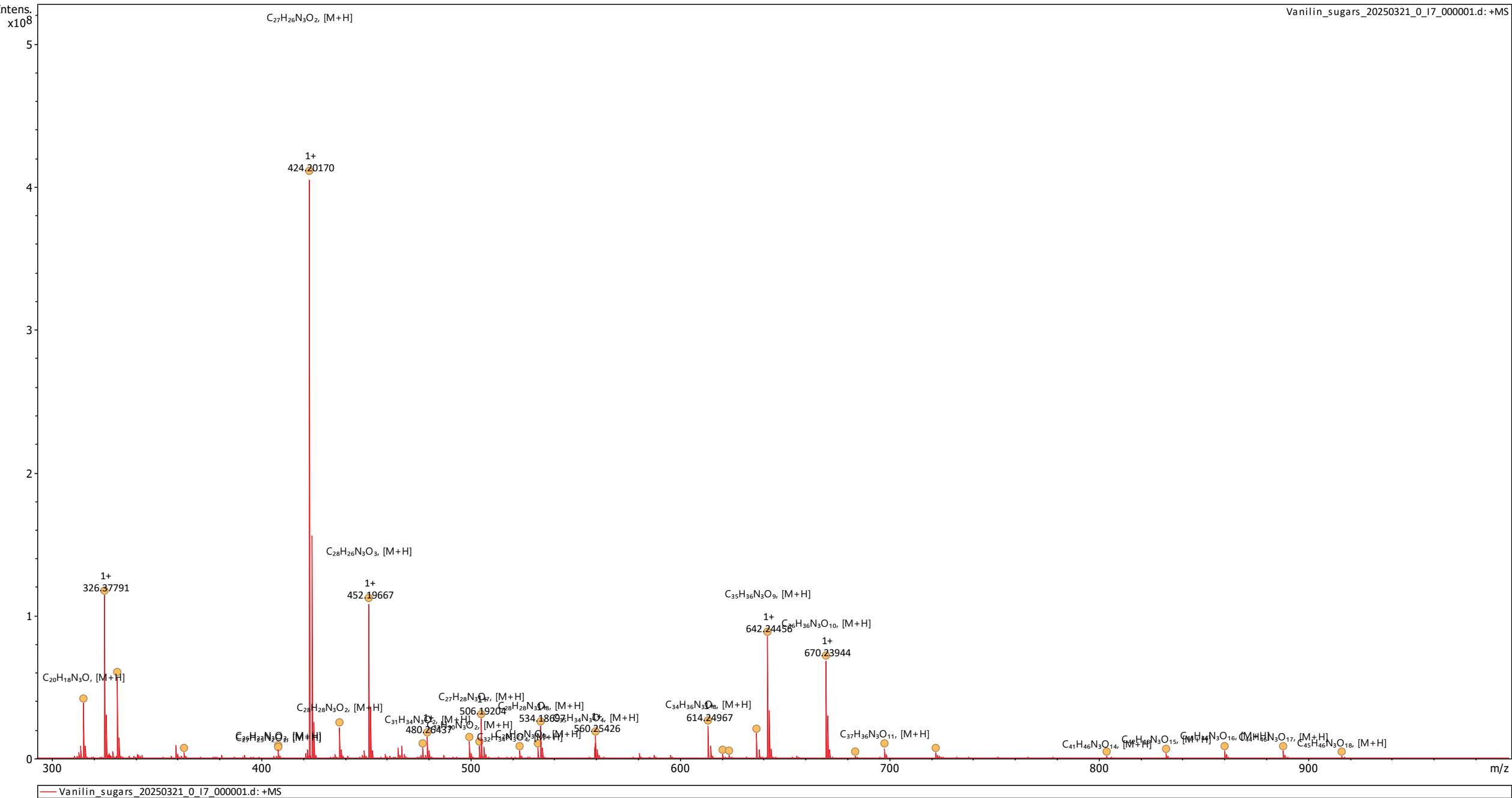

Xylose

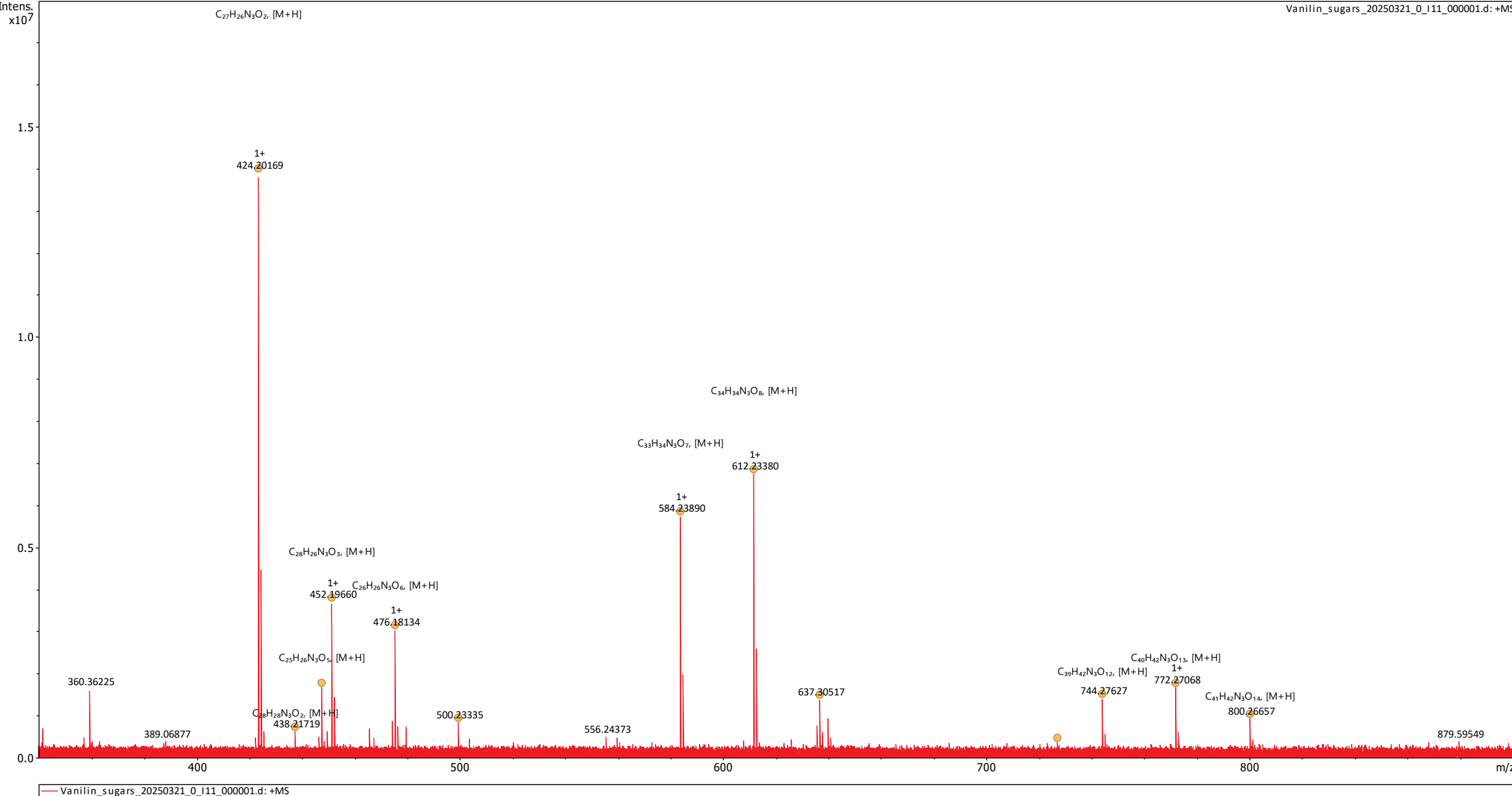

# Lactose

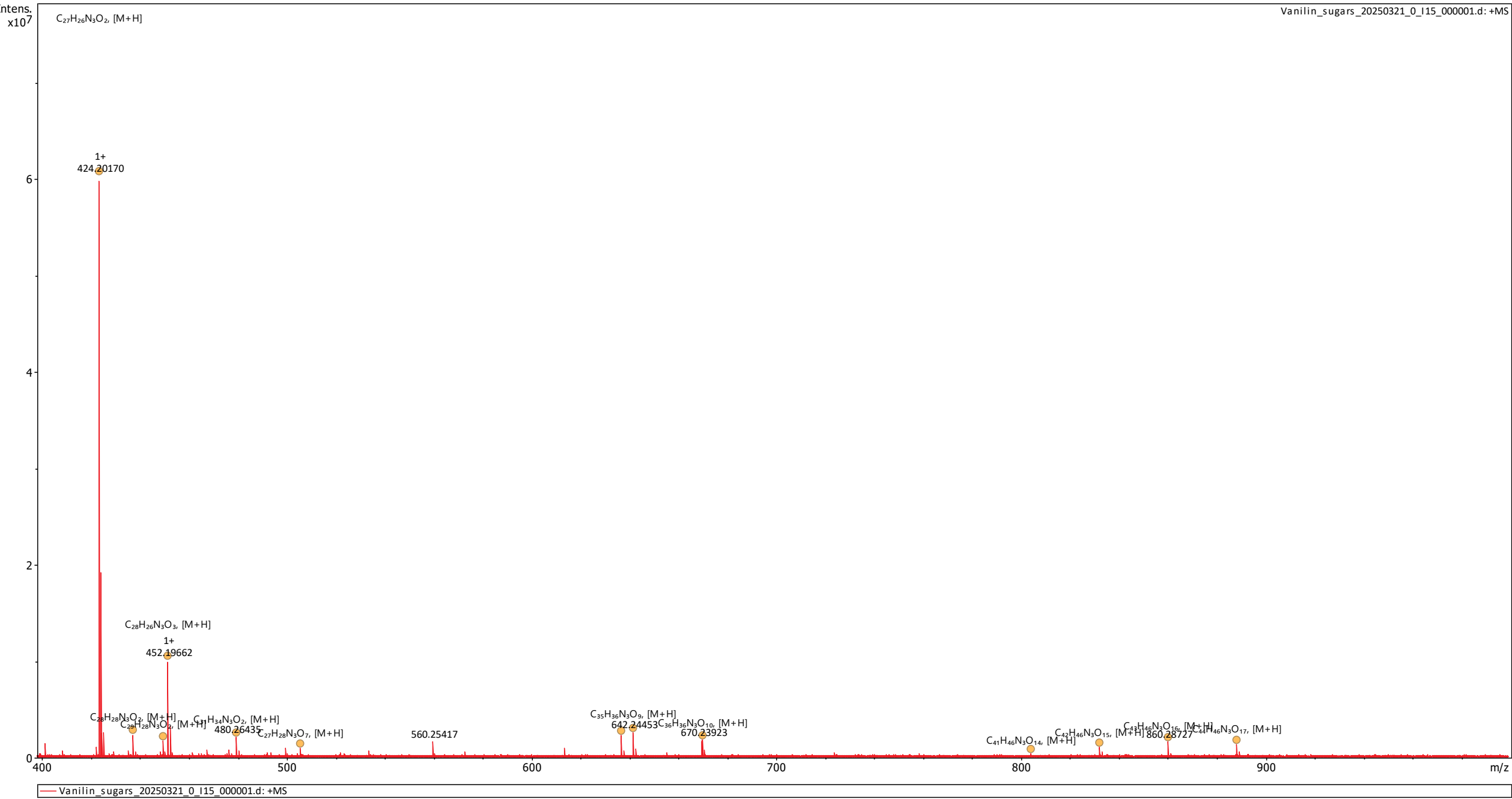

Supplement: Supplementary Figure 4 — Confirmation of esterified D-glucose, D-xylose, L-fucose, and lactose using 15T solariX XR FT-ICR mass spectrometer (Bruker Daltonics). [file DataSheet4.pdf]
