# Supplementary material for: Mass spectrometric profiling of microbial polysaccharides using laser desorption/ionization – time-of-flight (LDI-TOF) and liquid chromatography-mass spectrometry (LC-MS): a novel method for structural fingerprinting and derivatization
Source: Front Cell Infect Microbiol. 2025 Oct 3;15:1658802. doi: 10.3389/fcimb.2025.1658802 (PMC12531147; doi:10.3389/fcimb.2025.1658802)

CF PLUS CHEMICALS

CF Plus Chemicals s.r.o
Karásek 1767/1
62100 Brno
Česko
Tel.: +420 606 117 375
Email: sales@cfplus.cz

Cerfitikát analýzy

IUPAC jméno: 4-((4-aminophenyl)(4-((4-hydroxy-3-methoxybenzyl)amino)phenyl)methylene)cyclohexa-2,5-dien-1-iminium salt, adduct with 1,4-dihydrodicyanodichlorohydroquinone
Synonyma: Vanillyl p-rosanilin, addukt s redukovaným DDQ
Identifikátor šarže: CFAG01_45
CAS number: N/A
Chemický sumární vzorec: $C_{27}H_{26}N_3O_2$ (vzorec materčného kationtu)
Molekulová hmotnost: 424,52 g/mol (matečný kation)
Datum měření: 28.01.2025

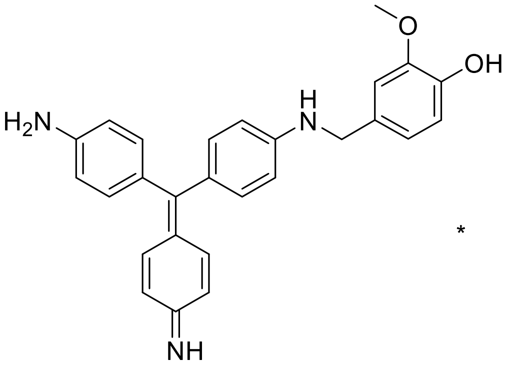

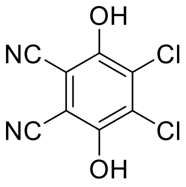

Molecular Weight: 229,02

Chemical Formula: $C_{27}H_{25}N_3O_2$
Molecular Weight: 423,52

Test	Specifikace	Výsledek
**Vzhled**	Fialová až černá krystalická látka	Fialová až černá krystalická látka
**NMR čistota**	95%+	vyhovuje
**HPLC analýza**		
**HRMS analýza**		

Příloha

Anotace spektra

^{1}H NMR (500 MHz, DMSO, 25 °C) δ 8.88 (s, 1H), 7.56 (s, 1H), 7.06 (ddd, *J* = 19.0, 16.9, 8.1 Hz, 11H), 6.81 (dd, *J* = 10.1, 6.3 Hz, 4H), 6.74 (d, *J* = 8.0 Hz, 1H), 4.48 (d, *J* = 6.2 Hz, 2H), 3.77 (s, 3H)

Pohled na celkové spektrum

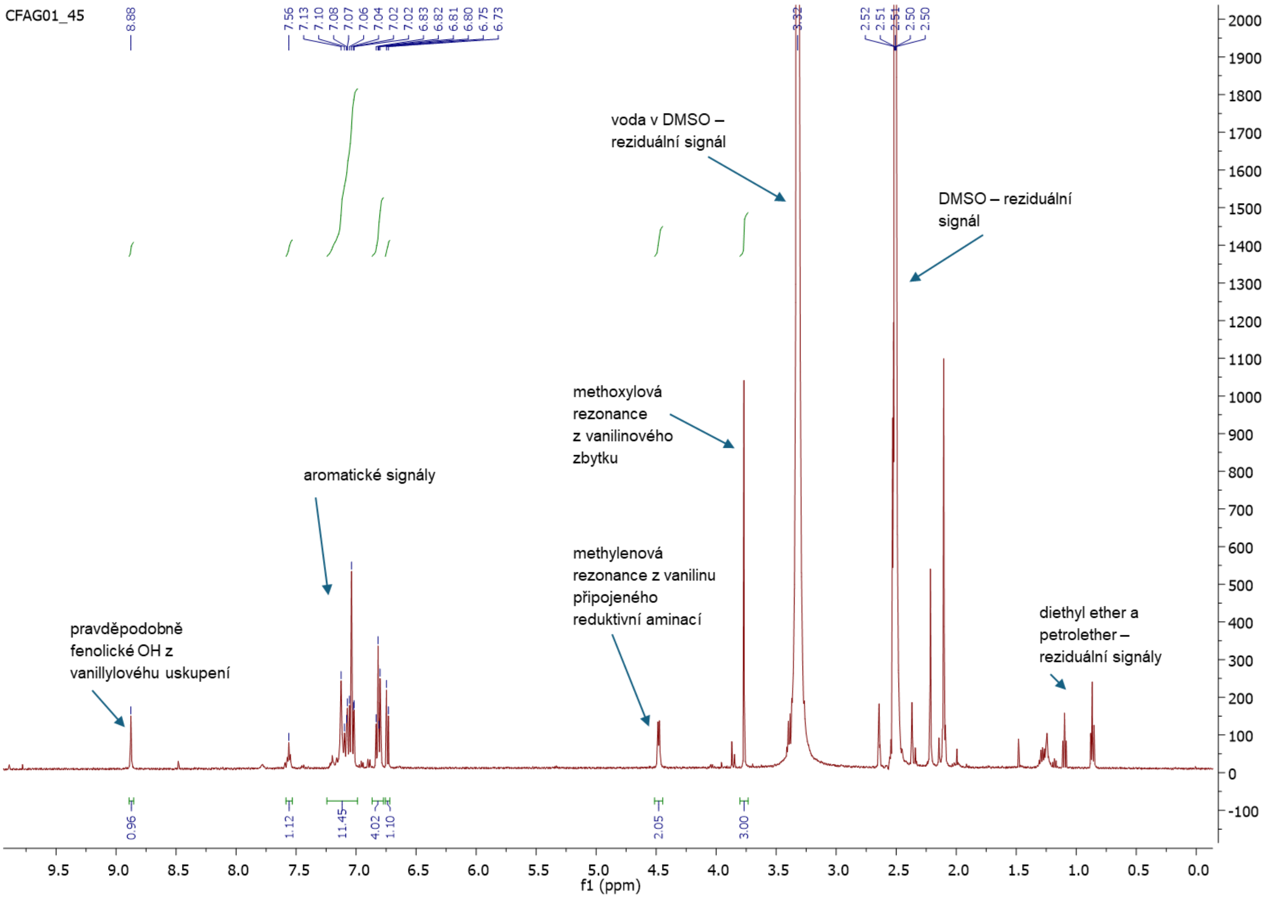

CF PLUS CHEMICALS

CF Plus Chemicals s.r.o
Karásek 1767/1
62100 Brno
Česko
Tel.: +420 606 117 375
Email: sales@cfplus.cz

Pohled na alifatickou část vanillylových rezonancí

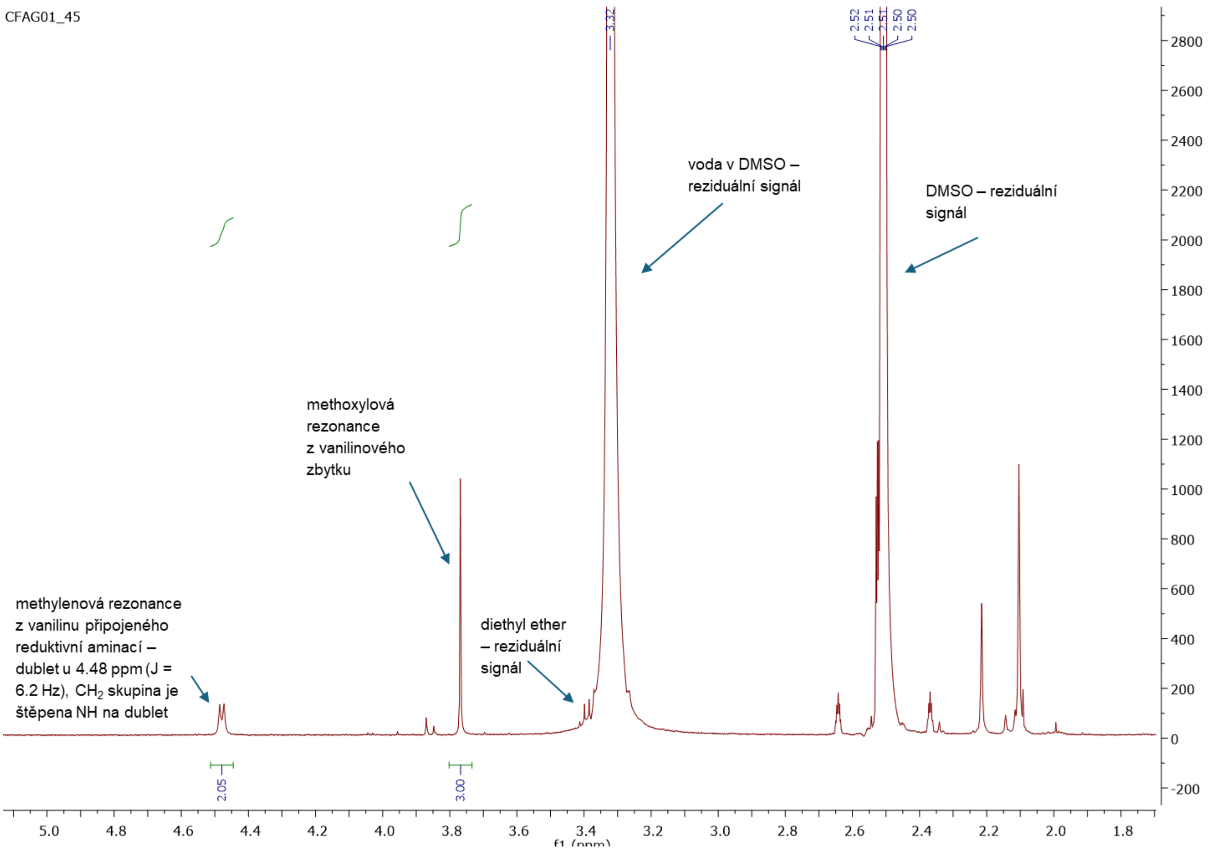

CF PLUS CHEMICALS

CF Plus Chemicals s.r.o
Karásek 1767/1
62100 Brno
Česko
Tel.: +420 606 117 375
Email: sales@cfplus.cz

Pohled na aromatickou část spektra molekuly

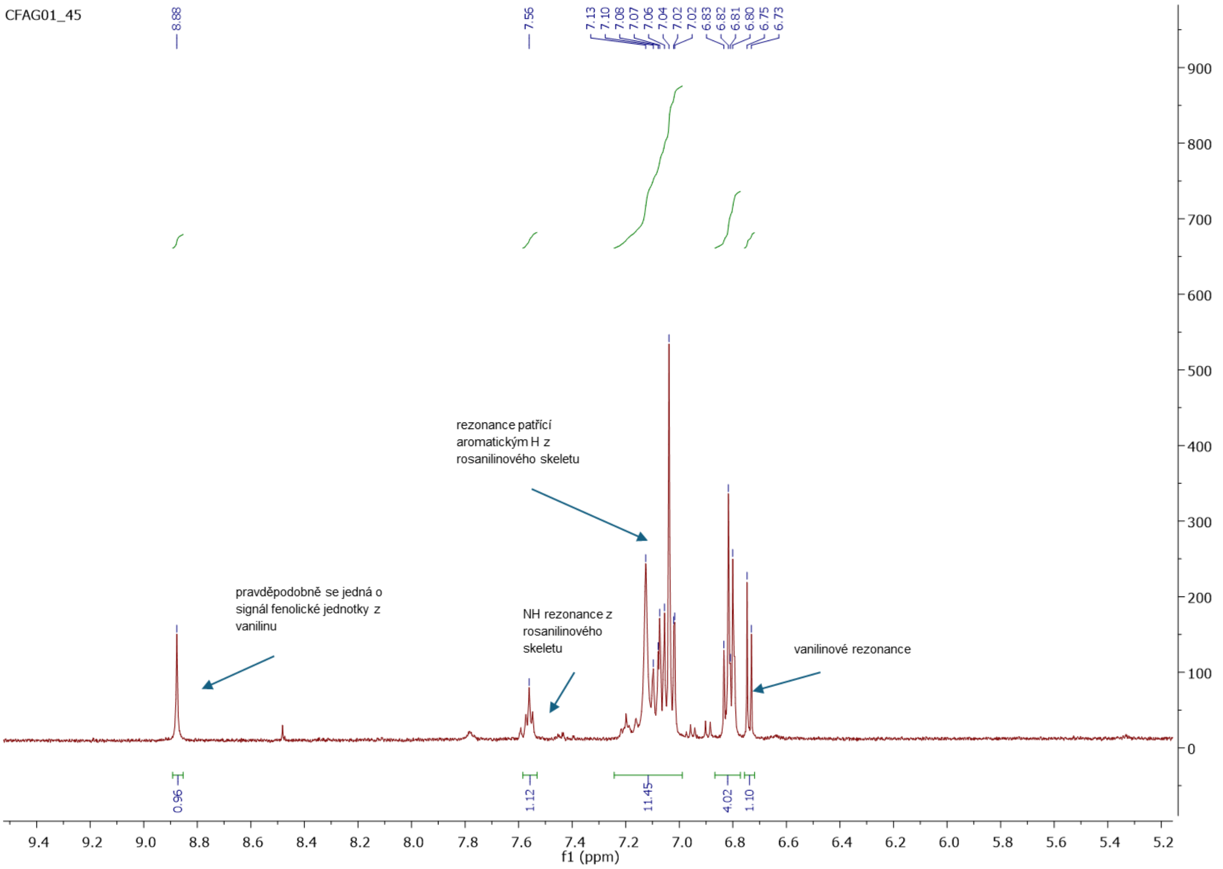

Supplement: Supplementary Figure 3 — Quality control protocol (NMR spectra) of commercially prepared Vanillyl-Pararosaniline (HD) ligand. [file DataSheet3.pdf]
